# A masked generative graph representation learning framework empowering precise spatial domain identification

**DOI:** 10.1093/bioinformatics/btag333

**Published:** 2026-05-22

**Authors:** Chuyao Wang, Tongdong Zhang, Hang Sun, Zhipeng Wu, Shuo Liang, Xueting Wang, Meirong Du, Yanchun Liang, Xin Gao, Qi Tang, Dong Xu, Xiaoyue Feng, An Zeng, Renchu Guan

**Affiliations:** Key Laboratory of Symbolic Computation and Knowledge Engineering of the Ministry of Education, College of Computer Science and Technology, Jilin University, Changchun, 130012, China; State Key Laboratory of Cell Biology, Center for Excellence in Molecular Cell Science, Shanghai Institute of Biochemistry and Cell Biology, Chinese Academy of Sciences, University of Chinese Academy of Sciences, Shanghai, 200031, China; Key Laboratory of Symbolic Computation and Knowledge Engineering of the Ministry of Education, College of Computer Science and Technology, Jilin University, Changchun, 130012, China; Key Laboratory of Symbolic Computation and Knowledge Engineering of the Ministry of Education, College of Computer Science and Technology, Jilin University, Changchun, 130012, China; Key Laboratory of Symbolic Computation and Knowledge Engineering of the Ministry of Education, College of Computer Science and Technology, Jilin University, Changchun, 130012, China; Key Laboratory of Symbolic Computation and Knowledge Engineering of the Ministry of Education, College of Computer Science and Technology, Jilin University, Changchun, 130012, China; Shanghai Key Laboratory of Maternal Fetal Medicine, Shanghai First Maternity and Infant Hospital, School of Medicine, Tongji University, Shanghai, 201204, China; Key Laboratory of Symbolic Computation and Knowledge Engineering of the Ministry of Education, College of Computer Science and Technology, Jilin University, Changchun, 130012, China; Zhuhai Sub Laboratory of Key Laboratory of Symbolic Computation and Knowledge Engineering of Ministry of Education, Zhuhai College of Science and Technology, Zhuhai, 519041, China; King Abdullah University of Science and Technology, Mathematical and Computer Science and Engineering Division, King Abdullah University of Science and Technology (KAUST), Thuwal, 23955-6900, Saudi Arabia; State Key Laboratory of Cell Biology, Center for Excellence in Molecular Cell Science, Shanghai Institute of Biochemistry and Cell Biology, Chinese Academy of Sciences, University of Chinese Academy of Sciences, Shanghai, 200031, China; Department of Electrical Engineering and Computer Science, Bond Life Sciences Center, University of Missouri, Columbia, MO 65211, United States; Key Laboratory of Symbolic Computation and Knowledge Engineering of the Ministry of Education, College of Computer Science and Technology, Jilin University, Changchun, 130012, China; State Key Laboratory of Cell Biology, Center for Excellence in Molecular Cell Science, Shanghai Institute of Biochemistry and Cell Biology, Chinese Academy of Sciences, University of Chinese Academy of Sciences, Shanghai, 200031, China; Key Laboratory of Symbolic Computation and Knowledge Engineering of the Ministry of Education, College of Computer Science and Technology, Jilin University, Changchun, 130012, China

## Abstract

**Motivation:**

Spatial transcriptomics (ST) enables the measurement of gene expression while preserving the spatial context of tissues. However, the sparsity of ST data leads to poor usage of gene expression and spatial information, resulting in the embeddings that are not well represented and challenging for downstream analyses.

**Results:**

Here, we introduced GSG, a generative self-supervised representation learning framework for ST data that leverages a masking mechanism to learn informative representations. For spatial domain identification, GSG consistently outperformed state-of-the-art methods across benchmarking datasets, regardless of sequencing platforms. In addition, we applied GSG to an in-house human fetal heart dataset, revealing anatomically coherent spatial domains and identifying *APCDD1* as an endocardial-specific marker potentially involved in congenital heart disease. Our results showcase GSG’s superiority and underscore its valuable contributions to advancing ST analysis.

**Availability and Implementation:**

Our software package is available at https://github.com/keaml-Guan/GSG.

## Graphical Abstract

**Graphical Abstract:**
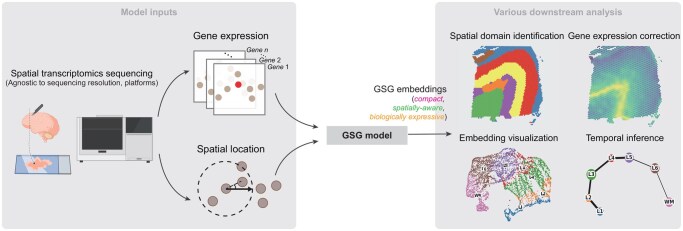

## 1 Introduction

Spatial transcriptomics (ST) enables gene expression profiling while preserving spatial context, providing crucial insights into cellular interactions and microenvironmental organization. Existing ST technologies can be broadly divided into imaging-based and sequencing-based approaches. While, imaging-based approaches can achieve single-cell resolution (Shah *et al.* 2016), they typically offer limited gene coverage (Liu *et al.* 2026). In contrast, sequencing-based approaches provide transcriptome-wide coverage, but are often constrained by the physical size of the capture units (Ji *et al.* 2020). Moreover, these data are typically sparse and noisy, presenting challenges that become increasingly pronounced at higher resolutions, where fewer molecules are captured per spatial unit (Chen *et al.* 2022).

A fundamental task in the analysis of such data is spatial domain identification, which aims to partition cells or spots into spatially coherent regions based on similarities in their transcriptional profiles. This process is essential for characterizing tissue heterogeneity. Existing methodologies can be broadly categorized into conventional machine-learning and statistical frameworks, as well as deep-learning-based approaches.

Traditional machine-learning and statistical frameworks integrates spatial and gene expression information by leveraging the transcriptional similarity among neighboring spots. For instance, BayesSpace (Zhao *et al.* 2021) employs a fully Bayesian clustering method to encourage spatially proximal spots to occupy the same cluster. Similarly, stLearn (Pham *et al.* 2023) incorporates spatial coordinates and histological features to normalize gene expression, utilizing the Louvain algorithms for initial clustering and subsequently refining these into subclusters based on physical proximity. While effective in certain context, these methods generally rely on linear or shallow representations as the foundation for downstream analyses. Consequently, they possess a limited capacity to capture complex, nonlinear transcriptomic architectures and the intricate coupling between molecular profiles and their spatial context.

Deep-learning-based methods leverage neural networks to learn nonlinear representations of ST data, significantly advancing spatial domain identification. For instance, SpaGCN (Hu *et al.* 2021) integrates gene expression, spatial location, and histology information within a graph convolutional framework (GCN), employing a deep embedding clustering (DEC) module for domain identification. More recently, self-supervised graph learning strategies have been introduced to enhance extraction, which can be further categorized into reconstruction-based and contrastive-learning-based approaches. Reconstruction-based methods, such as SEDR (Xu *et al.* 2024), STAGATE (Dong and Zhang, 2022), DeepST (Xu *et al.* 2022), and STMGAMF (Fu *et al.* 2025), learn latent representations by recovering features through variational or graph autoencoder frameworks. However, these objectives often emphasize exact, point-wise feature reconstruction, rendering them sensitive to the noise and dropout inherent in ST data. Furthermore, when neighborhood aggregation and reconstruction objectives are tightly coupled, these methods are often prone to over-smoothing, which can obscure fine-grained biological boundaries.

In contrast, contrastive-learning-based approaches (e.g., CCST (Li *et al.* 2022), SpaceFlow (Ren *et al.* 2022), and GraphST (Long *et al.* 2023]) learn discriminative embeddings by maximizing agreement between different views or samples. While promising, their performance frequently depends on the manual construction of positive/negative pairs and data augmentation strategies, which can introduce training instability and significant computational overhead particularly in sparse ST data. Crucially, despite their success, most existing methods were not explicitly designed to address sparsity and dropout as a primary modeling challenge.

To address these limitations, we propose GSG, a masked Generative Self-supervised Graph learning framework for spatial domain identification in ST data. The design of GSG is fundamentally motivated by the inherent sparsity and noise of ST data, coupled with the observation that spatial domain identification relies on the assumption of local homophily, where spatially adjacent spots typically share similar tissue regions or microenvironmental contexts. Within this setting, compelling the model to infer masked features from surrounding ST context provides a naturally robust objective for representation learning.

Furthermore, given that ST data are frequently compromised by dropout, our masking mechanism serves as a principled simulation of incomplete molecular observations. This encourages the model to mitigate over-reliance on potentially corrupted raw input of individual spots, instead prioritizing the extraction of more resilient representations from contextual information. To prevent the prediction task from collapsing into a trivial autoencoding-style reconstruction, we introduce a re-masking mechanism at the embedding level, which curtails a direct feature leakage to the decoder. Finally, recognizing that masking removes a substantial portion of input information, we argue that point-wise reconstruction objectives (e.g., mean square error (MSE)) are ill-suited for exact value recovery and prone to instability. We therefore adopt the Scaled Cosine Error (SCE) loss, which prioritizes the directional consistency of expression vectors over exact entry-wise magnitudes. This approach is inherently more compatible with heavily masked, noisy, and sparse ST data, providing greater stability against outliers compared to conventional MSE.

To benchmark the effectiveness of GSG, we compared it with existing methods on various ST datasets generated by different platforms, such as Visium, Slide-seqV2, and Stereo-seq. Our results demonstrate that GSG outperforms existing methods across various downstream tasks, including identifying spatial domains, inferring spatial trajectories, and learning biologically informative representations for high-quality data visualization.

## 2 Materials and methods

### 2.1 Overview of GSG

**Figure 1:**
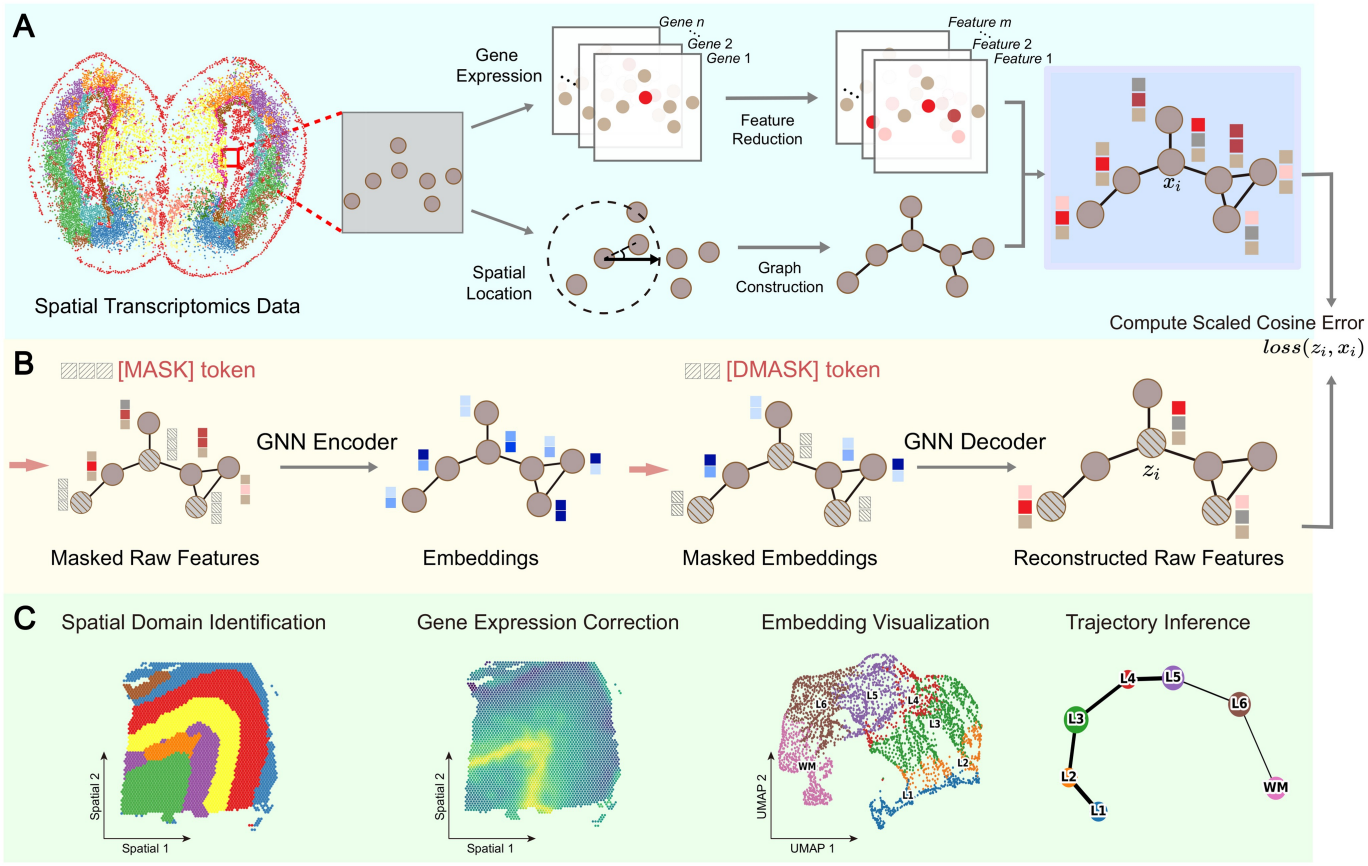
Overview of GSG. (A) The input ST data consists of gene expression and spatial location, which can be reformulated as a graph. Specifically, the spatial information is used to build the adjacency structure of the graph, and the gene expression information preprocessed by PCA reduction or HVG selection serves as the node features in the graph. (B) In the encoding phase, GSG first randomly selects a subset of nodes, and masks their features with a token [MASK]. Then a GNN encodes the gene expression information and the structure information into the cell embeddings. In the decoding phase, GSG re-masks the embeddings of selected nodes with another token [DMASK]. Then, GSG employs a GNN as the decoder to reconstruct the input node features of the masked nodes. The GNN and the trainable tokens are optimized by minimizing the scaled cosine error between the raw features $x_{i}$ and the reconstructed feature $z_{i}$ of each node. (C) The output of GSG can be directly applied for many downstream tasks, e.g., spatial domain identification, gene expression correction, embedding visualization, and trajectory inference.

Inspired by the success of BERT (Devlin *et al.* 2019) and GraphMAE (Hou *et al.* 2022), which leverage masked generative self-supervised learning for representation learning, we developed GSG, a generative self-supervised graph learning framework with a masking mechanism. Specifically, GSG treats ST data as a graph data, where each spot is modeled as a node, spatial proximity in physical space defines the graph topology, and gene expression profiles serve as node features (Fig. 1A). Although sequencing units vary across platforms (*e.g.*, cell, pixel, or bin), we use the term “spot” throughout for simplicity. GSG then performs masked self-supervised graph learning, allowing spatially neighboring spot features to be more effectively integrated when learning representations for each spot (Fig. 1B). The resulting spatially informed embeddings can be seamlessly applied to a variety of downstream analyses (Fig. 1C), such as spatial domain identification and pseudo‐temporal trajectory inference.

### 2.2 ST data preprocessing

The ST data contain two components: gene expression and spatial location information. GSG takes these data as input and outputs spatially coherent graph representations for each spot. According to the biological assumption that cells influence their neighbors according to the diffusion principle, an adjacency graph can be constructed using spatial coordinates. The gene expression profile of each spot is then used as the node features. Therefore, ST data can be formulated as a graph G=(V,A,X), where V denotes the node (spot) set, *A* the adjacency matrix, and *X* the node feature matrix.

To obtain the adjacency matrix *A*, GSG first calculates the distance matrix between spots based on their physical position. Then GSG chooses a predefined threshold *r*. If the distance between spot *i* and spot *j* is less than *r*, then $A_{i}{\;}_{j}=1$; otherwise, $A_{i}{\;}_{j}=0$. For all data, we determined the size of *r* based on the average of 6–10 neighbors around each spot point. Although a predefined radius can result in different numbers of neighbors for different cells, this variation may itself reflect biologically meaningful tissue architecture, as local cell density is an important aspect of spatial heterogeneity. Moreover, from the perspective of cell-cell communication, defining neighbors within a fixed physical radius is more biologically interpretable than enforcing a fixed number of neighbors. We also provide a KNN-based graph construction interface in the GSG package for users who wish to explore alternative neighborhood definitions. In particular, we recommend using KNN when local density heterogeneity is more likely to arise from technical artifacts rather than biological structure, such as tissue damage or uneven section quality.

To acquire the node feature *X*, we adopted two commonly used data preprocessing strategies for gene expression, following standard practices in ST data analysis. One is based on principal component analysis (PCA) implemented in Scanpy (Wolf *et al.* 2018) package. Specifically, the raw gene expression data undergo filtering, normalization, and scaling, and the high-dimensional gene features are further reduced by PCA to 600 principal components. The other is based on highly variable gene (HVG) selection. The raw gene expression data is filtered, normalized and log-transformed, and then top 3000 HVGs are chosen as the node features.

### 2.3 GSG graph encoder with masking mechanism

GSG first applies a uniform random sampling strategy without replacement to select nodes to be masked. Let ρ denote the masking ratio, i.e., defined as the proportion of nodes sampled into the masked set $\tilde{V}$. Its empirical effect is further analyzed in the sensitivity analysis presented in the Results section and Supplementary Materials, available as supplementary material at *Bioinformatics* online. In Graph Neural Networks (GNNs), a node’s representation is inherently contingent upon its local neighborhood. To maintain balanced learning signals, we employ a random sampling strategy that mitigates edge cases where a node’s neighborhood becomes either entirely occluded or fully exposed. Specifically, we sample a subset of nodes $\tilde{V}\subset V$ and replace their feature vectors with a trainable mask token [MASK], represented as $x_{\left[M\right]}\in R^{d}$. Formally, the masked feature matrix $\tilde{X}=\text{MASK}(X)$ is constructed such that the feature vector $\tilde{x_{i}}$ for each node $v_{i}\in V$ is defined as:


$$\tilde{x_{i}}=\{\begin{array}{cc} x_{\left[M\right]}, & v_{i}\in \tilde{V}\\ x_{i}, & v_{i}\notin \tilde{V} \end{array}$$

The encoder module of GSG takes the masked features matrix $\tilde{X}$ and the adjacency matrix *A* as inputs. It integrates spatial context from neighboring spots and captures feature dependencies to generate a latent representation for each spot. Let $f_{E}$ denote the graph encoder and $H\in R^{N\times d_{h}\;}$ represent the resulting embedding matrix. The encoding process is formulated as follows:


$$H=f_{E}(A,\tilde{X})$$

### 2.4 GSG graph decoder with re-masking mechanism

Prior to the decoding phase, the latent embeddings *H* undergo a re-masking procedure. Specifically, the embeddings corresponding to the originally masked nodes $\tilde{V}$ are replaced with a distinct decoding mask token [DMASK], represented as a learnable vector $h_{\left[M\right]}\in R^{d_{h}}$. This re-masking operation, denoted as $\tilde{H}=\text{REMASK}(H)$, yields modified embeddings where each $\tilde{h_{i}}$ is formally defined as:


$$\tilde{h_{i}\;}=\{\begin{array}{cc} h_{\left[M\right]}, & v_{i}\in \tilde{V}\\ h_{i} & v_{i},\notin \tilde{V} \end{array}$$

Then, in contrast to GSG graph encoder module, the decoder aims to reconstruct the original node features from the re-masked latent embeddings $\tilde{H}$. The graph decoder, denoted as $f_{D}$, takes the modified embeddings $\tilde{H}$ and the adjacency matrix *A* as input to recover the feature matrix. The decoding process is formulated as follows:


$$Z=f_{D}(A,\tilde{H})$$

Where $Z\in R^{N\times d}$ denotes the reconstructed feature matrix, representing the recovered attributes of the nodes.

### 2.5 Loss function

The training objective is to reconstruct the masked features of nodes in $\tilde{V}$. Considering a substantial fraction of the input information is removed by masking, point-wise reconstruction objectives such as MSE make exact recovery of the absolute value at each feature dimension difficult and potentially unstable. We therefore adopt the SCE loss to emphasizes consistency in the overall direction of the expression vector rather than exact entry-wise magnitude, making it more suitable for heavily masked inputs. In addition, compared with MSE, which can be more easily influenced by outliers, SCE tends to be more stable in noisy settings, making it particularly advantageous for ST data (Hou *et al.* 2022).

Given an original feature *X* and reconstructed output *Z*, the SCE loss function can be defined as following, here γ is a scaling hyperparameter that can be tuned across different datasets. Mechanistically, this factor acts as an adaptive sample reweighting mechanism, directing the model’s focus toward challenging samples to improve.


$$L_{\mathrm{SCE}}=\frac{1}{\left|\tilde{V}\right|}\sum_{v_{i}\in \;\tilde{V}}{\left(1-\frac{x_{i}{\;}^{T}\;z_{i}}{\left\|x_{i}\right\|\left\|z_{i}\right\|}\right)}^{\gamma },\;\;\;\;\gamma \geq 1$$

After training, the learned embedding *H* yields a compact, spatially aware, and biologically expressive representation for each spot. These embeddings can be seamlessly integrated into various downstream analyses, such as spatial domain identification and pseudo–temporal trajectory inference. Furthermore, the reconstructed matrix *Z* serves as an enhanced gene expression profile, offering refined data for subsequent exploration. Comprehensive details regarding the downstream analyses conducted in this study are provided in Supplementary Text 1, available as supplementary material at *Bioinformatics* online.

### 2.6 Implementation details

GSG employs a multi-layer graph isomorphism networks (Xu *et al.* 2019) as the graph encoder. For all datasets evaluated in this study, the hidden dimension was maintained at 128. The number of GNN layers was set to 2 for the Visium mouse brain dataset and 3 for all other datasets. Regarding activation functions for the spatial domain identification task, PReLU was utilized for the Slide-seq V2 dataset, while ELU was applied to the remaining datasets. For the gene expression correction task, to mitigate the potential over-smoothing effects associated with increased GNN depth, we implemented a streamlined Multi-Layer Perceptron (MLP) as the decoder instead of a deep GNN structure.

The GSG model was optimized using the Adam optimizer with a learning rate of 0.001 and a weight decay of 0.0002. Training epochs was 850 on the Slide-seq V2 mouse hippocampus dataset and 500 on all other datasets. All implementation details are summarized in Supplementary Text 2 and Supplementary Table 1, available as supplementary material at *Bioinformatics* online.

## 3 Results

### 3.1 GSG enhances spatial domain identification in the human DLPFC dataset

**Figure 2:**
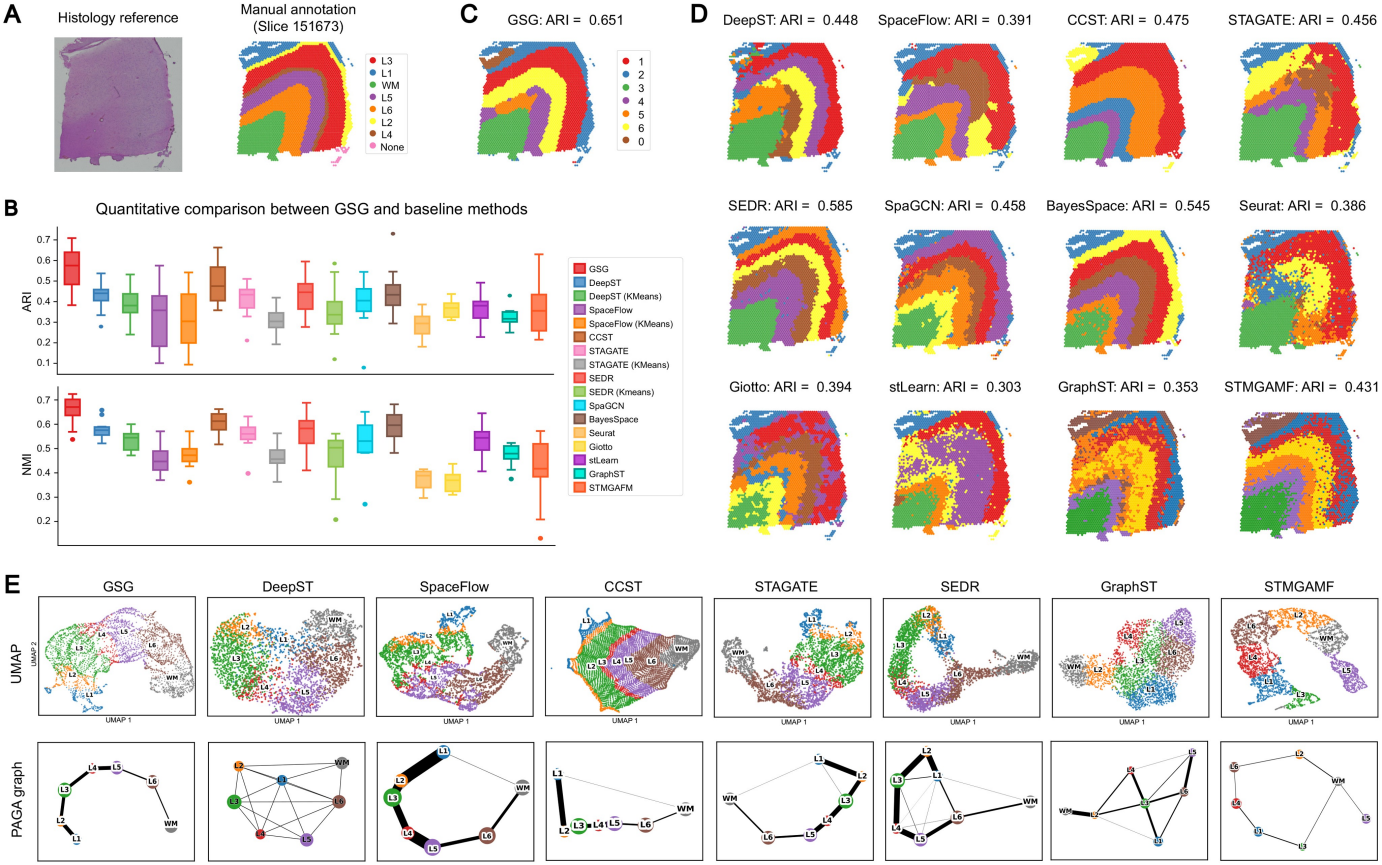
GSG enhances spatial domain identification in the human DLPFC dataset. (A) Histology reference and manual annotation of slice 151673 in DLPFC dataset. (B) Boxplot of ARI and NMI scores of GSG and 12 comparison methods across 12 slices. The center line, box limits, and whiskers represent the median, upper, and lower quartiles, and 1.5× interquartile range, respectively. (C) Spatial domains delineated by GSG of slice 151673. (D) Spatial domains delineated by comparison methods on slice 151673. € UMAP visualizations and PAGA trajectory of the embeddings generated by GSG and other methods’ embeddings on slice 151673.

To assess the effectiveness of GSG embedding, we first employed it to delineate spatial domains. The learned spatially-informed embeddings were clustered via K-means to identify tissue regions. We utilized the human dorsolateral prefrontal cortex (DLPFC) dataset (Maynard *et al.* 2021) as a benchmark, consisting of 12 sections sequenced on the Visium platform. Each section’s manual annotations (L1–L6 and WM) served as the gold standard for evaluation (Fig. 2A).

For spatial domain identification, we compared GSG with eight deep learning-based methods (DeepST, SpaceFlow, CCST, STAGATE, SEDR, SpaGCN, GraphST, and STMGAMF) and four traditional machine learning-based methods (BayesSpace, Seurat (Hao *et al.* 2021), Giotto (Dries *et al.* 2021), and stLearn). Implementation details are provided in Supplementary Text 3, available as supplementary material at *Bioinformatics* online, where we specified that recommended settings from original publications were used for all baselines. To ensure a fair comparison, we standardized the spatial domain identification by applying a consistent clustering algorithm (K-means) to the embeddings extracted by all applicable methods, including GSG, DeepST, SpaceFlow, STAGATE, SEDR, GraphST, and STMGAMF. Given that adjusted Rand index (ARI) and Fowlkes–Mallows index (FMI) evaluate clustering performance through pairwise co-assignment, while normalized mutual information (NMI) offers a distinct information-theoretic perspective (Supplementary Text 4, available as supplementary material at *Bioinformatics* online), performance was quantitatively evaluated using the three metrics. And GSG consistently achieved the best overall performance on this dataset, illustrated in Fig. 2B and Supplementary Fig. 1, available as supplementary material at *Bioinformatics* online. For instance, GSG achieved the highest average ARI across the 12 slices, reaching 0.554. Notably, when all embedding-based methods were coupled with the same K-means clustering procedure, the advantage of GSG became even more pronounced, indicating that GSG learned higher-quality embeddings for spatial domain identification.

Taking slice 151673 as an example, GSG accurately identified spatial domains with an ARI of 0.651, outperforming existing state-of-the-art methods (Fig. 2C, D). Visual comparison further showed that GSG produced sharper and more clearly defined cortical boundaries. To demonstrate that GSG can extract biologically expressive spot embeddings, we performed uniform manifold approximation and projection (UMAP) and partition-based graph abstraction (PAGA) trajectory inference on the embeddings generated by GSG and other comparison methods (Fig. 2E). UMAP analysis showed that GSG produced distinct clusters, grouping spots from the same layers together with clear boundaries. By contrast, CCST-based clustering yielded more dispersed clusters, and DeepST clustering exhibited blurred inter-layer boundaries. PAGA trajectory analysis further demonstrated that GSG separated each layer more clearly and recovered a continuous developmental trajectory from L1 to L6 and WM. Notably, the trajectories inferred from GSG embeddings were consistent with the established “inside-out” cell migration model of cortical development (Gilmore and Herrup 1997), whereas trajectories derived from embeddings generated by other methods often contained redundant edges, indicating weaker biological relevance. Together, these results (Supplementary Figs. 2–12, available as supplementary material at *Bioinformatics* online) underscore the strong biological significance captured by GSG embeddings.

### 3.2 GSG refines gene expression profiles to better characterize spatial patterns

We hypothesized that GSG could leverage spatial neighborhood context through its masking mechanism during GNN learning to denoise ST data and amplify biologically relevant signals. To validate this, we applied GSG to a DLPFC sample (slice 151673) and compared original gene expression profiles with their denoised counterparts. Layer-specific gene enrichment was analyzed via violin plots to quantify the noise-reduction effects using manual annotations from Maynard *et al.* as the ground-truth reference (Fig. 2A).

**Figure 3:**
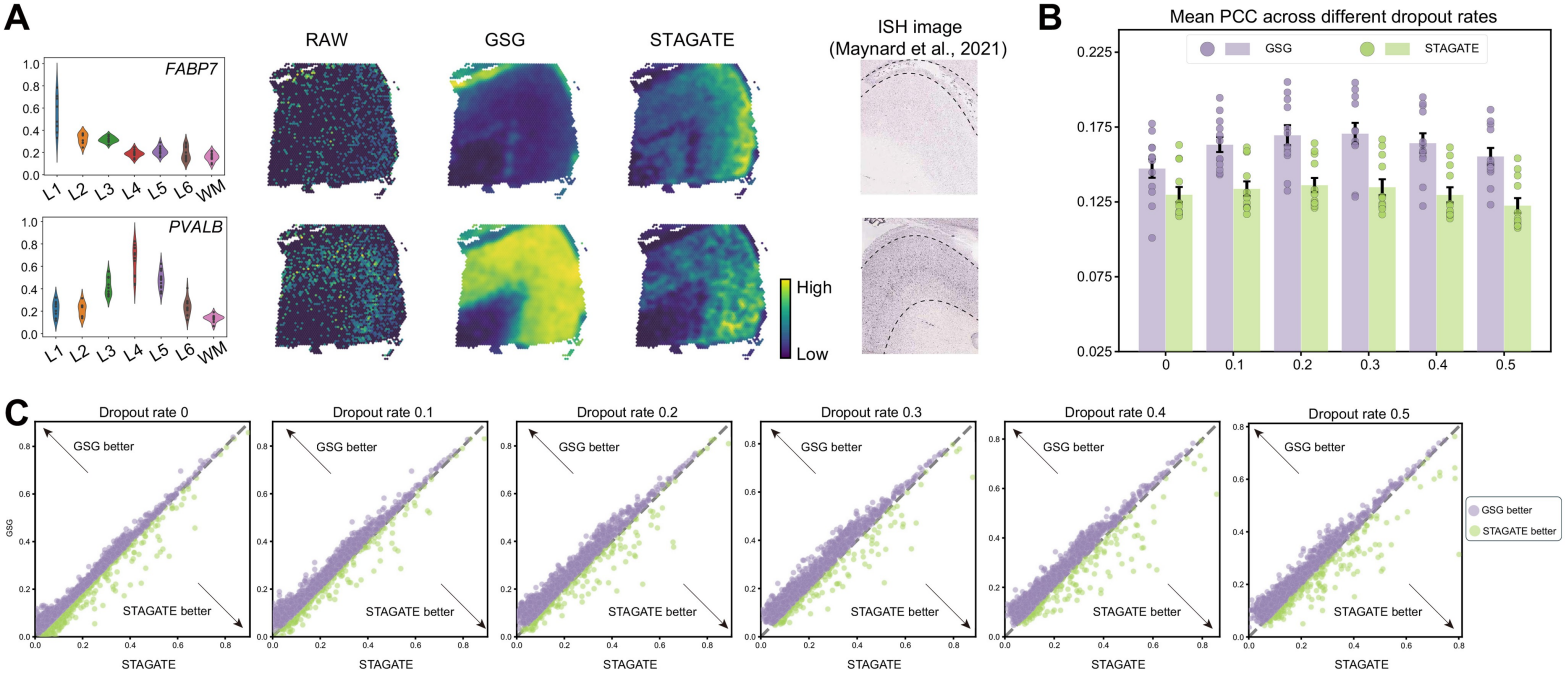
GSG refines gene expression profiles to better characterize spatial patterns. (A) Left: Violin plots showing layer-specific enrichment of *FABP7* and *PVALB*. Middle: Spatial expression patterns derived from the original measured data, GSG-corrected data, and STAGATE-corrected data. Right: Corresponding ISH images provided as ground-truth references. (B) Mean PCC between corrected and measured gene expression across 12 DLPFC slices under various simulated dropout rates. Each dot represents the mean PCC for an individual slice, while bar heights indicate the average of these slice-level means. Error bars represent the standard error of the mean. (C) Scatter plot of the PCC value between measured and corrected gene expression generated by GSG and STAGATE on slice 151673. Each dot represents an individual gene.

The results showed that, for instance, *FABP7* and *PVALB*, which are selectively expressed in layers L1 and L4 respectively, exhibited significantly sharper enrichment within their target layers following GSG correction. Meanwhile, background signals in non-target regions were substantially attenuated (Fig. 3A). In contrast, the denoising effect achieved by STAGATE was less pronounced, further highlighting GSG’s superior spatial specificity. Additional representative examples are provided in Supplementary Fig. 13, available as supplementary material at *Bioinformatics* online.

To systematically evaluate the recovery of dropout-corrupted signals, we simulated varying dropout rates across all 12 DLPFC slices by randomly masking a defined fraction of non-zero (Zhou and Wu, 2025) expression values. Recovery performance was quantified using the Pearson correlation coefficient (PCC) between the recovered and original expression profiles. Across all tested dropout rates, GSG consistently outperformed STAGATE across the 12 slices (Fig. 3B). Gene-level PCC comparisons (Fig. 3C) further demonstrated that GSG excels for the majority of genes, with the performance gap becoming more pronounced at higher dropout rates (Supplementary Fig. 14, available as supplementary material at *Bioinformatics* online). Furthermore, GSG-corrected expression faithfully recapitulated the original spot-to-spot similarity structure (Supplementary Fig. 15, available as supplementary material at *Bioinformatics* online). Collectively, these results demonstrate GSG’s capacity to enhance signal quality for downstream spatial analyses.

### 3.3 GSG demonstrates robust generalization across diverse tissue types and ST platform

To further demonstrate the generalizability of GSG, we evaluated it on datasets generated by ST platforms with single-cell or near single-cell resolution, including Slide-seqV2, Stereo-seq, and seqFISH.

Regarding the Visium breast cancer analysis, the dataset’s high intra tumoral heterogeneity—comprising four primary morphotypes (healthy, tumor edge, ductal carcinoma *in situ*/lobular carcinoma *in situ* (DCIS/LCIS), and invasive ductal carcinoma (IDC)) and 20 fine-grained spatial domains—presented a significant challenge for spatial characterization. On this benchmark, GSG achieved a leading ARI score of 0.632, demonstrating its ability to not only maintain spatial continuity but also faithfully recapitulate the complex histological architecture (Supplementary Fig. 16A, available as supplementary material at *Bioinformatics* online). Specifically, GSG provided clearer and more precise partitioning of critical regions, such as DCIS/LCIS 1 and IDC 4, outperforming existing methods.

The robustness of GSG was further evidenced in its application to a Visium coronal mouse brain section (Supplementary Fig. 16B, available as supplementary material at *Bioinformatics* online). Although manual annotations were not provided for this dataset, we used the Allen Mouse Brain Atlas as a reference. GSG precisely delineated major structural landmarks, including the visual cortex (domains 4, 11, 13), olfactory cortex (domain 8), caudate-putamen (CNU, domain 12), thalamus (domain 15), and hypothalamus (domain 14). Notably, GSG faithfully reconstructed the prototypical arch-shaped morphology of the hippocampus, with specific clusters successfully distinguishing the dentate gyrus (domain 1) from Ammon’s horn (domain 3). Beyond spatial clustering, we evaluated how GSG-mediated feature recovery impacts gene expression profiles. Following correction, marker genes exhibited enhanced spatial coherence and a significantly improved signal-to-noise ratio. For instance, the expression of *AK5* (typically localized to the olfactory areas, cortical subplate, and hippocampal formation) and *AGT* (enriched in the hypothalamic lateral zone and sensory–motor-related thalamic regions) was fragmented or nearly absent in the raw data due to severe dropout. Following GSG correction, these expression patterns were refined into continuous and biologically consistent structures. This transformation demonstrates the efficacy of GSG in recovering missing transcripts and purifying spatial signals.

The vastly increased spatial resolution of these single-cell and near-single-cell platforms presents a significant computational challenge, as cell counts often exceed those of Visium spots by orders of magnitude. To evaluate GSG’s performance in these high-resolution contexts, we first analyzed the Slide-seqV2 mouse hippocampus dataset (Stickels *et al.* 2021). In this dataset of over 40,000 cells, GSG accurately delineated key anatomical regions and cell types, such as the CA1, CA2, CA3, Subiculum, and Dentate Pyramids, showing high concordance with the ground truth (Supplementary Fig. 16C, available as supplementary material at *Bioinformatics* online).

The versatility of GSG was further confirmed on a Stereo-seq mouse olfactory bulb dataset (Chen *et al.* 2022) (Supplementary Fig. 16D, available as supplementary material at *Bioinformatics* online). The spatial domains identified by GSG exhibited a clear hierarchical organization that precisely matched manual anatomical annotations. Specifically, moving from the inner to the outer layers, GSG captured the rostral migratory stream (domain 9), the granule cell layer (domain 7), the internal plexiform layer (domain 6), the mitral cell layer (domain 5), the external plexiform layer (domain 2), the glomerular layer (domain 1), and the olfactory nerve layer (domains 3 and 4).

Finally, we validated GSG in a low-throughput scenario using a seqFISH mouse embryo dataset (Lohoff *et al.* 2022), which measures only 387 genes (Supplementary Fig. 16E, available as supplementary material at *Bioinformatics* online). Despite the limited gene count, GSG accurately clustered major embryonic regions, including the erythroid cells, splanchnic mesoderm, and the gut tube. Collectively, these results underscore the robustness and versatility of GSG in identifying spatial domains across a wide spectrum of tissue architectures and sequencing technologies.

### 3.4 GSG deciphers the spatial architecture and lineage-specific markers of the developing human heart

**Figure 4:**
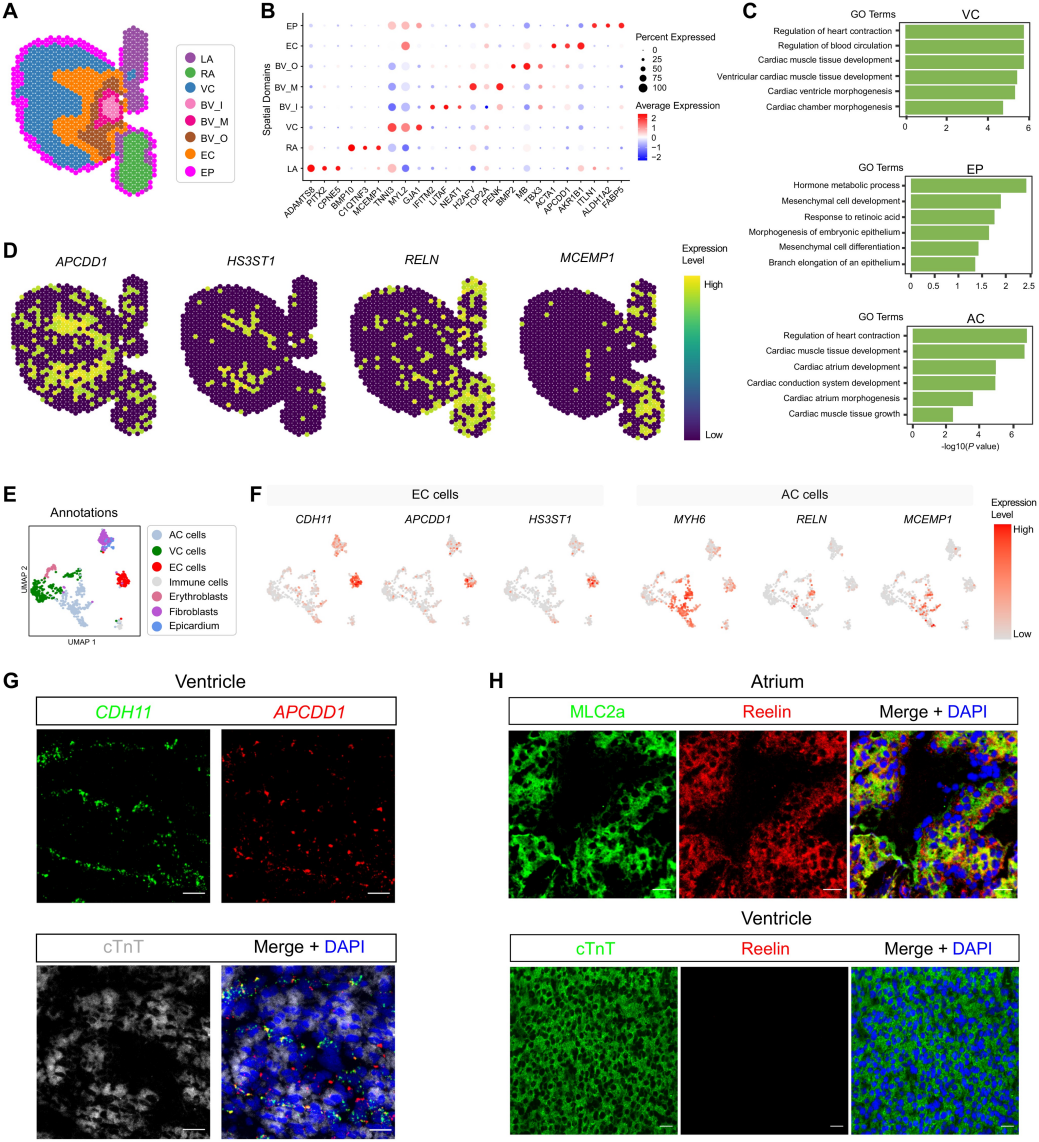
GSG deciphers the spatial architecture and lineage-specific markers of the developing human heart. (A) Spatial domains in ST data of human fetal heart detected by GSG. (B) Dot plot shows characteristic gene expression profiles in different spatial domains. (C) GO terms enriched in VC, EP, and AC domains. (D) Spatial feature plot shows that *APCDD1* and *HS3ST1* are mainly expressed in domain EC, while *RELN* and *MCEMP1* are specifically expressed in domain AC. (E) Single-cell RNA sequencing dataset of fetal human hearts from 9–13 weeks of gestation. Unsupervised clustering and manual annotation identified seven major cell populations. (F) Feature plot on UMAP of the marker gene expression of EC and AC cells. (G) FISH and immunofluorescence staining experiments show that *APCDD1* is mainly expressed in *CDH11* ${\;}^{+}$ endocardium cells, but not cTnT${\;}^{+}$ cardiomyocytes. (H) Immunofluorescence staining experiments show that Reelin is mainly expressed in MLC2a${\;}^{+}$ atrial myocardial tissue, but not in cTnT${\;}^{+}$ cardiomyocytes from the ventricles.

The intricate anatomical architecture and diverse cellular composition of the human heart present a formidable challenge for conventional ST analysis methods. To evaluate GSG’s capacity for resolving spatial domains within such complex tissues, we applied the framework to an in-house Visium dataset derived from a healthy human fetal heart at nine weeks of gestation. GSG successfully delineated seven primary spatial domains, including the atrial cardiomyocyte (AC)-enriched, ventricular cardiomyocyte (VC)-enriched, endocardium (EC), and epicardium (EP) domains, alongside three distinct layers of the vasculature: blood vessel inner (BV_I), middle (BV_M), and outer (BV_O) (Fig. 4A). Notably, GSG further resolved the AC domain into two finer sub-domains corresponding to the left and right atrium (LA and RA). These identified domains exhibited sharp spatial boundaries and were characterized by highly distinct gene expression signatures (Fig. 4B and Supplementary Fig. 17A, available as supplementary material at *Bioinformatics* online).

Functional enrichment analysis revealed that marker genes within the VC domain were significantly associated with Gene Ontology (GO) biological processes such as “regulation of heart contraction” and “ventricular cardiac muscle tissue development.” These findings indicate that the VC domain plays a critical role in embryonic ventricular morphogenesis (Fig. 4C, top). Similarly, the EP domain was enriched for terms including “hormone metabolic process,” “response to retinoic acid,” and “morphogenesis of embryonic epithelium,” underscoring the regulatory influence of hormones and retinoic acid on epicardial development (Fig. 4C, middle). Furthermore, marker genes in the AC domain were primarily involved in “cardiac atrium development” and “cardiac conduction system development” (Fig. 4C, bottom). Collectively, these results demonstrate that GSG accurately identifies functionally distinct spatial domains within the human fetal heart. To explore the intercellular dynamics of these regions, we performed spatial domain communication network analysis using CellChat (Jin *et al.* 2021). This analysis revealed that the blood vessel and epicardium domains serve as the predominant signaling hubs, acting as the primary sources of molecular cues within the developing fetal heart (Supplementary Fig. 17B, available as supplementary material at *Bioinformatics* online).

To investigate the transcriptional regulatory landscape underlying these spatial domains, we employed SCENIC (Aibar *et al.* 2017) to identify domain-specific transcription factor activities, or regulons (Supplementary Fig. 17C, available as supplementary material at *Bioinformatics* online). This analysis revealed that the atrium and ventricle domains are governed by distinct regulatory programs, which likely orchestrate their divergent developmental trajectories. Beyond known regulators, we identified several novel domain-specific candidates potentially involved in fetal heart maturation. Specifically, the EC domain exhibited predominant expression of *APCDD1* and *HS3ST1*, while the AC domain was characterized by the enrichment of *RELN* and *MCEMP1* (Fig. 4D).

To ensure the robustness of these findings, we first cross-validated these spatial expression patterns using high-resolution scRNA-seq data from fetal human hearts spanning 9–13 weeks of gestation. This analysis confirmed the cell-type-specific enrichment of our newly identified markers, showing that *APCDD1* and *RELN* are predominantly expressed within the EC and AC populations, respectively (Fig. 4E and Supplementary Fig. 17D–F, available as supplementary material at *Bioinformatics* online). Notably, while previous studies (Asp *et al.* 2019) provided initial spatial maps of the 9-week human embryo heart, the limited data quality hindered the detection of these refined signals (Supplementary Fig. 18, available as supplementary material at *Bioinformatics* online). To provide definitive spatial evidence, we conducted RNA-fluorescence in situ hybridization (FISH) and immunofluorescence staining. We confirmed that *APCDD1* transcripts are specifically localized to *CDH11*-positive endocardial cells (Fig. 4F), while the Reelin protein (encoded by *RELN*) is exclusively expressed in MLC2a-positive atrial myocardium (Fig. 4G).

The biological significance of these markers is underscored by their potential clinical relevance to congenital heart disease (CHD). Notably, patients with left ventricular noncompaction (LVNC) have been reported to harbor single-nucleotide variants in the *APCDD1* gene (Siguero-Álvarez *et al.* 2023). Given that endocardial cells play a crucial role in supporting early myocardial trabeculation and compact myocardial growth, developmental abnormalities in this lineage often result in LVNC (Liu *et al.* 2023). Our findings suggest a potential mechanistic link where *APCDD1* mutations contribute to the pathogenesis of CHD by inducing developmental defects within the human embryonic endocardium.

In conclusion, by leveraging the high-resolution analytical capabilities of GSG, our study reveals previously unreported, spatially restricted genes in the fetal heart that are linked to developmental defects. These findings highlight the potential of GSG as a powerful tool for discovering novel biological insights and identifying potential therapeutic targets within complex spatial transcriptomic landscapes.

### 3.5 Ablation study and sensitivity analyses

We conducted systematic ablation analyses to evaluate the contributions of key architectural components within GSG. Specifically, we compared the full GSG model against three ablated variants: (1) no-MASK, in which excludes the masking mechanism, thereby reducing GSG to a basic graph autoencoder; (2) no-ReMASK, in which lacks the re-masking mechanism; and (3) no-SCE, in which the SCE loss was replaced with MSE.

**Figure 5:**
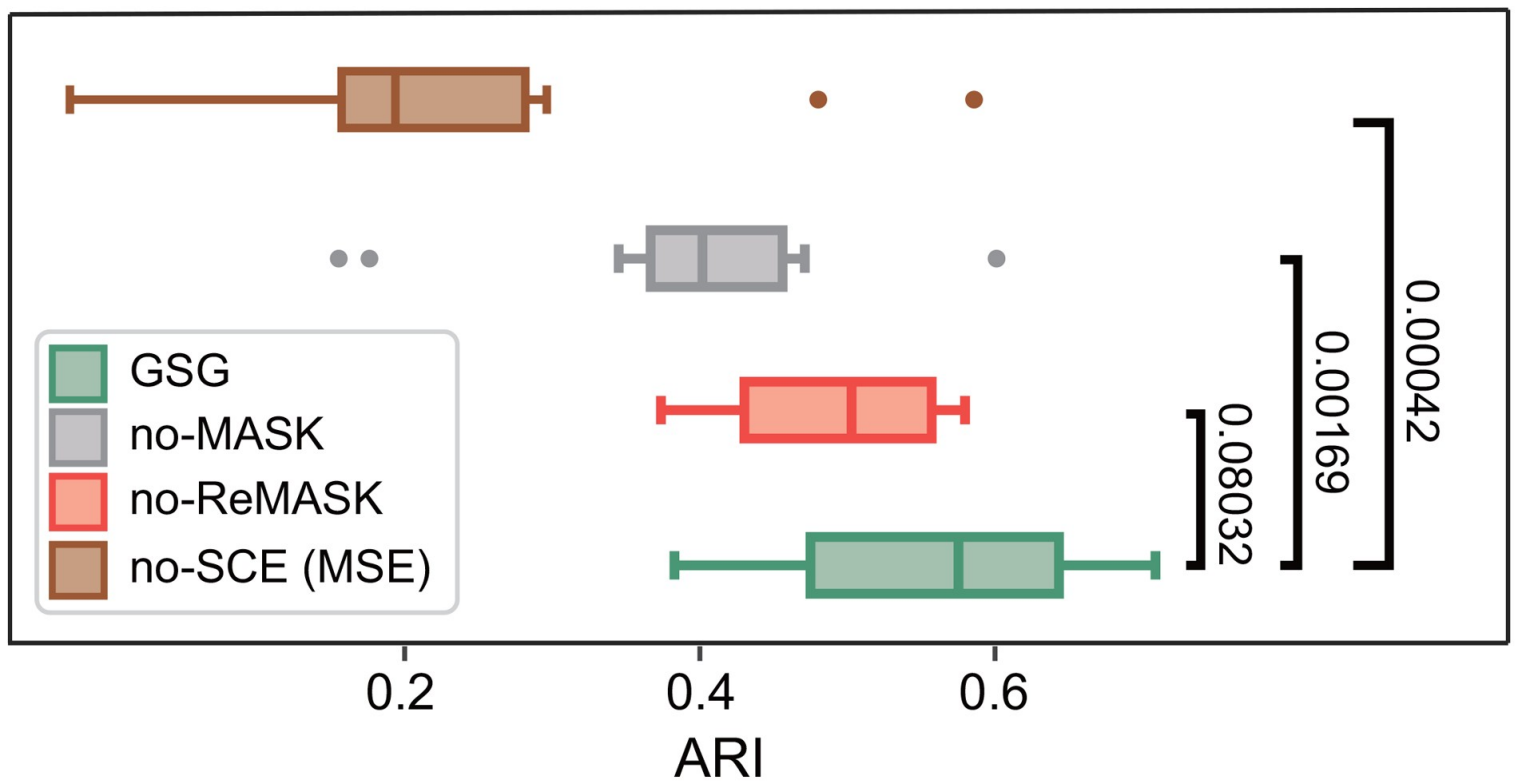
Ablation study of GSG design components. Boxplots illustrate the ARI values for spatial domain identification on the DLPFC dataset, comparing the full GSG model with its three ablated variants. In each boxplot, the center line denotes the median, the box limits represent the upper and lower quartiles, and the whiskers extend to 1.5× the interquartile range.

As shown in Fig. 5, the full GSG model consistently achieved higher ARI scores across all 12 slices of the DLPFC dataset compared to the three ablated variants. Among these components, the masking mechanism constitutes the core of GSG, as it explicitly compels the model to infer the features of a target node from its surrounding context. In this framework, re-masking and SCE loss serve as complementary designs that enhance the efficacy of masked reconstruction. The re-masking mechanism is introduced to mitigate potential information leakage from target nodes during the decoding phase. Without re-masking, the decoder might partially exploit target-node representations, shifting the task toward direct self-reconstruction and thereby undermining the intended contextual prediction objective.

Furthermore, the computational efficiency of all compared methods is quantitatively evaluated in Supplementary Text 6, available as supplementary material at *Bioinformatics* online, where we also systematically examine the relationship between the masking ratio and computational overhead. Our analysis reveals that while the time overhead of GSG increases near-linearly with the number of masked nodes, the marginal cost of reconstruction remains exceptionally low compared to the encoding phase. Consequently, GSG achieves a favorable balance between model complexity and scalability, even when a substantial fraction of the input data is masked.

We next assessed the sensitivity of GSG to the masking ratio parameter. GSG showed relatively stable performance across a wide range of masking ratios (0.2–0.9) (Supplementary Figs. 19 and 20, available as supplementary material at *Bioinformatics* online). A more comprehensive analysis of parameter sensitivity, including graph construction strategies and neighborhood radius, is presented in Supplementary Text 5, available as supplementary material at *Bioinformatics* online. In addition, the computational efficiency of all compared methods is quantitatively evaluated in Supplementary Text 6, available as supplementary material at *Bioinformatics* online, where we also examine the relationship between masking ratio and computational cost. Overall, the time overhead of GSG increases approximately linearly with the number of masked nodes.

## 4 Discussion

In this study, we present GSG, a generative graph representation learning framework specifically engineered for spatial transcriptomics. The architecture of GSG is predicated on two defining characteristics of ST data: the inherent sparsity and noise within high-dimensional expression matrices, and the critical dependence of spatial domain identification on local tissue context. By formulating representation learning as a contextual feature recovery task, GSG encourages the model to infer incomplete molecular signals from spatial neighborhoods. This approach effectively shifts the focus from relying on potentially biased, directly observed values to a more robust, neighborhood-aware reconstruction.

The integration of graph-based context aggregation with a multi-stage masking strategy—comprising masking, remasking, and an SCE-based objective—allows GSG to extract biologically informative representations. These embeddings are directly applicable to a variety of downstream tasks. Here, we primarily focused on spatial domain identification, demonstrating both qualitatively and quantitatively that GSG yields superior biological coherence compared to existing methods. Beyond clustering, GSG-derived embeddings facilitate more resolved trajectory inference through PAGA, while its reconstruction capability effectively mitigates dropout-related noise—a feature essential for gene-level spatial pattern analysis. Crucially, the robust performance of GSG across diverse species, tissue types, and sequencing platforms underscores its broad utility in the ST field.

Beyond its computational performance, the biological utility of GSG is most notably demonstrated by its application to the developing human heart. While previous efforts were limited by data noise (Asp *et al.* 2019), GSG successfully resolved a refined spatial map, enabling the identification of four novel markers with lineage-specific expression patterns. The discovery of *APCDD1* as a spatially restricted marker in the endocardium is particularly significant. Given the known association between *APCDD1* mutations and left ventricular noncompaction (LVNC) (Siguero-Álvarez *et al.* 2023), our spatial evidence provides a critical developmental context that was previously missing. By precisely localizing *APCDD1* to the endocardial cells during the key window of trabeculation, GSG moves beyond simple clustering—it provides a structural basis for understanding how localized gene dysfunction might manifest as congenital defects. This case study underscores GSG’s potential as a discovery engine that can bridge the gap between high-dimensional spatial data and clinical pathology.

In conclusion, GSG offers a sophisticated masked graph learning paradigm tailored for the challenges of ST data. As spatial sequencing technologies continue to evolve toward higher resolution and larger scales, we anticipate that GSG will serve as a pivotal tool for uncovering previously elusive spatial structures and functionally relevant genes, ultimately deepening our understanding of tissue organization in health and disease.

## Supplementary Material

btag333_Supplementary_Data

## Data Availability

All datasets analyzed in this study are publicly available for download, with comprehensive descriptions provided in Supplementary Text 7, available as supplementary material at *Bioinformatics* online. Our in-house data: a healthy fetal human heart of 10X Visium ST dataset has been deposited in Gene Expression Omnibus (GEO) with accession number GSE231496. Detailed procedures regarding internal data preprocessing are documented in Supplementary Text 8, available as supplementary material at *Bioinformatics* online.

## Code availability

Source code is available at https://github.com/keaml-Guan/GSG. An archived version corresponding to this manuscript is available at https://doi.org/10.5281/zenodo.19303888.

## References

[btag333-B1] Aibar S , González-Blas CB, Moerman T et al SCENIC: single-cell regulatory network inference and clustering. Nat Methods 2017;14:1083–6. 28991892 10.1038/nmeth.4463 PMC5937676

[btag333-B2] Asp M , Giacomello S, Larsson L et al A spatiotemporal organ-wide gene expression and cell atlas of the developing human heart. Cell 2019;179:1647–6. 31835037 10.1016/j.cell.2019.11.025

[btag333-B3] Chen A , Liao S, Cheng M et al Spatiotemporal transcriptomic atlas of mouse organogenesis using DNA nanoball-patterned arrays. Cell 2022;185:1777–92. 35512705 10.1016/j.cell.2022.04.003

[btag333-B4] Devlin J , Chang M-W, Lee K et al Bert: Pre-training of deep bidirectional transformers for language understanding. In: *Proceedings of the 2019 conference of the North American chapter of the association for computational linguistics: human language technologies, volume 1* (long and short papers), p. 4171–86. Minneapolis, Minnesota: Association for Computational Linguistics, 2019.

[btag333-B5] Dong K , Zhang S. Deciphering spatial domains from spatially resolved transcriptomics with an adaptive graph attention auto-encoder. Nat Commun 2022;13:1739. 35365632 10.1038/s41467-022-29439-6 PMC8976049

[btag333-B6] Dries R , Zhu Q, Dong R et al Giotto: a toolbox for integrative analysis and visualization of spatial expression data. Genome Biol 2021;22:78. 33685491 10.1186/s13059-021-02286-2 PMC7938609

[btag333-B7] Fu Y , Nan M, Ren Q et al A multi-view graph convolutional network framework based on adaptive adjacency matrix and multi-strategy fusion mechanism for identifying spatial domains. Bioinformatics 2025;41:btaf172. 40233119 10.1093/bioinformatics/btaf172 PMC12041416

[btag333-B8] Gilmore EC , Herrup K. Cortical development: layers of complexity. Curr Biol 1997;7:R231–34. 9162498 10.1016/s0960-9822(06)00108-4

[btag333-B9] Hao Y , Hao S, Andersen-Nissen E et al Integrated analysis of multimodal single-cell data. Cell 2021;184:3573–87. 34062119 10.1016/j.cell.2021.04.048 PMC8238499

[btag333-B10] Hou Z , Liu X, Cen Y et al Graphmae: Self-supervised masked graph autoencoders. In: *Proceedings of the 28th ACM SIGKDD Conference on Knowledge Discovery and Data Mining*, New York, NY, USA: Association for Computing Machinery*,* 2022, p. 594–604.

[btag333-B11] Hu J , Li X, Coleman K et al SpaGCN: integrating gene expression, spatial location and histology to identify spatial domains and spatially variable genes by graph convolutional network. Nat Methods 2021;18:1342–51. 34711970 10.1038/s41592-021-01255-8

[btag333-B12] Ji AL , Rubin AJ, Thrane K et al Multimodal analysis of composition and spatial architecture in human squamous cell carcinoma. Cell 2020;182:497–514. 32579974 10.1016/j.cell.2020.05.039 PMC7391009

[btag333-B13] Jin S , Guerrero-Juarez CF, Zhang L et al Inference and analysis of cell-cell communication using CellChat. Nat Commun 2021;12:1088. 33597522 10.1038/s41467-021-21246-9 PMC7889871

[btag333-B14] Li J , Chen S, Pan X et al Cell clustering for spatial transcriptomics data with graph neural networks. Nat Comput Sci 2022;2:399–408. 38177586 10.1038/s43588-022-00266-5

[btag333-B15] Liu H , Duan R, He X et al Endothelial deletion of PTBP1 disrupts ventricular chamber development. Nat Commun 2023;14:1796. 37002228 10.1038/s41467-023-37409-9 PMC10066379

[btag333-B16] Liu Y , Wang C, Wang Z et al High-parameter spatial multi-omics through histology-anchored integration. Nat Methods 2026;23:373–86. 41407925 10.1038/s41592-025-02926-6

[btag333-B17] Lohoff T , Ghazanfar S, Missarova A ,et al Integration of spatial and single-cell transcriptomic data elucidates mouse organogenesis. Nat Biotechnol 2022;40:74–85. 34489600 10.1038/s41587-021-01006-2 PMC8763645

[btag333-B18] Long Y , Ang KS, Li M et al Spatially informed clustering, integration, and deconvolution of spatial transcriptomics with GraphST. Nat Commun 2023;14:1155. 36859400 10.1038/s41467-023-36796-3 PMC9977836

[btag333-B19] Maynard KR , Collado-Torres L, Weber LM et al Transcriptome-scale spatial gene expression in the human dorsolateral prefrontal cortex. Nat Neurosci 2021;24:425–36. 33558695 10.1038/s41593-020-00787-0 PMC8095368

[btag333-B20] Pham D , Tan X, Balderson B et al Robust mapping of spatiotemporal trajectories and cell–cell interactions in healthy and diseased tissues. Nat Commun 2023;14:7739. 38007580 10.1038/s41467-023-43120-6 PMC10676408

[btag333-B21] Ren H , Walker BL, Cang Z et al Identifying multicellular spatiotemporal organization of cells with SpaceFlow. Nat Commun 2022;13:4076. 35835774 10.1038/s41467-022-31739-w PMC9283532

[btag333-B22] Shah S , Lubeck E, Zhou W et al In situ transcription profiling of single cells reveals spatial organization of cells in the mouse hippocampus. Neuron 2016;92:342–57. 27764670 10.1016/j.neuron.2016.10.001 PMC5087994

[btag333-B23] Siguero-Álvarez M , Salguero-Jiménez A, Grego-Bessa J et al A human hereditary cardiomyopathy shares a genetic substrate with bicuspid aortic valve. Circulation 2023;147:47–65. 36325906 10.1161/CIRCULATIONAHA.121.058767

[btag333-B24] Stickels RR , Murray E, Kumar P et al Highly sensitive spatial transcriptomics at near-cellular resolution with slide-seqV2. Nat Biotechnol 2021;39:313–9. 33288904 10.1038/s41587-020-0739-1 PMC8606189

[btag333-B25] Wolf FA , Angerer P, Theis FJ. SCANPY: large-scale single-cell gene expression data analysis. Genome Biol 2018;19:15. 29409532 10.1186/s13059-017-1382-0 PMC5802054

[btag333-B26] Xu C , Jin X, Wei S et al Deepst: identifying spatial domains in spatial transcriptomics by deep learning. Nucleic Acids Res 2022;50:e131. 36250636 10.1093/nar/gkac901 PMC9825193

[btag333-B27] Xu H , Fu H, Long Y et al Unsupervised spatially embedded deep representation of spatial transcriptomics. Genome Med 2024;16:12. 38217035 10.1186/s13073-024-01283-x PMC10790257

[btag333-B28] Xu K , Hu W, Leskovec J et al How powerful are graph neural networks? In: *International Conference on Learning Representations*, New Orleans, LA, USA, 2019, p. 1–17.

[btag333-B29] Zhao E , Stone MR, Ren X et al Spatial transcriptomics at subspot resolution with BayesSpace. Nat Biotechnol 2021;39:1375–84. 34083791 10.1038/s41587-021-00935-2 PMC8763026

[btag333-B30] Zhou X , Wu H. scHiClassifier: a deep learning framework for cell type prediction by fusing multiple feature sets from single-cell Hi-C data. Brief. Bioinform 2025;26:bbaf009. 10.1093/bib/bbaf009 PMC11744636 39831891

