# Supplementary material for: A masked generative graph representation learning framework empowering precise spatial domain identification
Source: Bioinformatics. 2026 May 22;42(6):btag333. doi: 10.1093/bioinformatics/btag333 (PMC13263152; doi:10.1093/bioinformatics/btag333)
Supplement: btag333_Supplementary_Data [file btag333_supplementary_data.zip › 01-Jun-2026_064654_SupplementaryMaterials.pdf]

Supplementary information

A masked generative graph representation learning framework empowering precise spatial domain identification

Table of Contents

Supplementary Text 3

Downstream Analysis . . . 3

Model Details . . . 4

Benchmarking Details . . . 5

Evaluation Metrics . . . 8

Parameters Sensitivity Analysis . . . 10

Computational Efficiency Analysis . . . 11

Data availability . . . 12

Preprocessing of Internal Data . . . 13

Supplementary Tables 14

Table S1 . . . 14

Supplementary Figures 15

Figure S1 . . . 15

Figure S2 . . . 15

Figure S3 . . . 16

Figure S4 . . . 18

Figure S5 . . . 19

Figure S6 . . . 20

Figure S7 . . . 21

Figure S8 . . . 22

Figure S9 . . . 23

Figure S10 . . . 24

Figure S11 . . . 25

Figure S12 . . . 26

Figure S13 . . . 27

Figure S14 . . . 28

Figure S15 . . . 30

Figure S16 . . . 31

Figure S17 . . . 33

Figure S18 . . . 35

Figure S19 . . . 36

Figure S20 . . . 37

Figure S21 . . . 38

References 39

Supplementary Text

1. Downstream Analysis

Spatial Domain Identification

We used different clustering methods to identify the spatial domains based on the embedding, which was obtained by the GSG encoder after training. If the number of labels is given, we use the K-means cluster algorithm implemented using the sklearn [Pedregosa et al., 2011] package.

Gene Expression Correction

We used the HVG method to preprocess the gene expression data. Then, we use the processed data to train the GSG network. After training, the data were passed through the encoder and decoder, and the reconstructed gene expression values were treated as the GSG-imputed expression.

Embedding Visualization

We used uniform manifold approximation and projection (UMAP) [McInnes et al., 2018] implemented in the Scanpy [Wolf et al., 2018] package to visualize the embeddings produced by different methods. Specifically, a k-nearest neighbor graph (n_neighbors = 10) was constructed based on the similarity between embeddings, and the resulting graph was embedded into two dimensions using UMAP for visualization.

Trajectory Inference

We used the partition-based graph abstraction (PAGA) [Wolf et al., 2019] algorithm implemented in the Scanpy package to depict the spatial trajectory. For fairness, we set the same parameters for the different algorithms when performing trajectory inference.

2. Model Details

2.1 Graph Encoder and Decoder Architecture Details

Graph Encoder: The encoder takes the input graph features and encodes them into latent embeddings. It employs a graph isomorphism network (GIN) architecture.

Graph Decoder: The decoder takes the embeddings generated by encoder and recover the masked nodes' features. As respect to spatial domain identification task, the graph decoder is GIN. For the gene correction task, to mitigate the over-smoothing effect introduced by stacking GNN layers, GSG uses a lighter and simpler MLP as the decoder.

Activation function: For the spatial domain identification task, PReLU was used for the Slide-seq V2 dataset, and ELU was used for the remaining datasets. For the gene correction task, to mitigate the over-smoothing effect introduced by stacking GIN layers, we used a lighter and simpler multi-layer perceptron as the decoder. PReLU was applied to the hidden layers, while the output layer used no activation function so as to preserve the flexibility of reconstructing continuous expression values.

Hidden dimension: Embedding dimensions were set 128 on all experiments in the manuscript.

2.2 Training Parameters

Optimizer: Adam optimizer was used on all experiments in the manuscript.

Learning Rate: The learning rate for the model is set to 0.001 on all experiments in the manuscript.

Weight Decay: A weight decay of 2e-4 is applied to regularize the model and prevent overfitting on all experiments in the manuscript.

Max Epochs: Training epochs was 850 on the Slide-seq V2 mouse hippocampus dataset and 500 on all other datasets.

Loss Function: GSG uses Scaled Cosine Error (SCE) as the loss function in all experiments in this manuscript. For the spatial domain identification task, SCE is computed only on the masked nodes, as the goal is to enforce contextual prediction and encourage the model to infer missing features from surrounding spatial and transcriptional information. This design preserves the self-supervised masking objective and prevents the model from degenerating into trivial autoencoding-style reconstruction. For the gene correction task, in contrast, SCE is computed on all nodes to provide denser reconstruction supervision over the whole graph, thereby enhancing the model's ability to recover the full expression matrix and impute missing gene expression values.

A systematic summary is provided in Table S1.

3. Benchmarking Details

To benchmark domain-defined performance, we compare GSG with the Seurat [Hao et al., 2021], Giotto [Dries et al., 2021], stLearn [Pham et al., 2023], BayesSpace [Zhao et al., 2021], SpaGCN [Hu et al., 2021], SEDR [Xu et al., 2024], STAGATE [Dong and Zhang, 2022], CCST [Li et al., 2022], SpaceFlow [Ren et al., 2022] and DeepST [Xu et al., 2022] using LIBD human dorsolateral prefrontal cortex (DLPFC) ST data. All baseline methods were implemented using default or author-recommended parameters as specified in their original publications or official software packages (e.g., official tutorials for the DLPFC benchmark). For other datasets, we adopted configurations from the most similar sequencing technologies. The only adjustment across all experiments was the alignment of the number of output clusters with ground-truth annotations to ensure a consistent basis for comparison. No additional dataset-specific hyperparameter tuning was performed.

Seurat [Hao et al., 2021], the raw ST mRNA count data were preprocessed to remove low-quality genes and transformed to remove technical artefacts and normalize the data. The RunPCA function was applied to the top 50 principal components (PCs), and the FindNeighbors function was used to calculate the shared nearest neighbours (SNNs). Then, the Louvain clustering algorithm was used to identify clusters with the SNN networks. We tried clustering at different resolutions to obtain the same number of clusters as the ground truth layers.

Giotto [Dries et al., 2021], the raw ST mRNA count data and the spot positions were used as input. We used the filterGiotto and normalizeGiotto functions to filter the low-quality genes and spots and normalize the data. CalculateHVG and runPCA were applied to obtain HVG genes and use these genes to descend. Next, we used the createSpatialNetwork function to create a spatial network and obtain the domain-defined result.

stLearn [Pham et al., 2023], the raw ST mRNA count data and the spot positions were used as input. The count matrix input was read via the Read10X function in the stLearn package. We sequentially used the filter_genes, normalize_total, log1p, and run_pca functions to preprocess the data. Moreover, the histological image of the tissue is also preprocessed using the tiling and extract_features functions in the stLearn package. Then, the SME_normalize function was performed with the parameter settings of use_data = "raw" and weights = "physical_distance". Finally, the scale and run_pca were used on the normalized data with 30 principal components. The principal components from normalized data will then be used for the domain defined via Leiden.

BayesSpace [Zhao et al., 2021], we input the raw ST mRNA count expression matrix and the spot positions through the getRDS method. Next, we used the modelGeneVar and getTopHVGs methods in the scran method to model the variance of the log-expression profile of each gene and to extract the top 2000 highly variable genes. Then, the runPCA function in the scater package was used for principal components. The BayesSpace clustering method spatialCluster was performed with 15 principal components, with 50,000 MCMC iterations and gamma = 3 for smoothing.

SpaGCN [Hu et al., 2021], the raw ST mRNA counts were preprocessed to remove low-quality genes and choose special genes by the prefilter_gene and prefilter_specialgenes functions in the SpaGCN python package sequentially. Then, the data were normalized and log-transformed. Next, the spot positions were used to calculate the adjacent matrix by the calculate_adj_matrix function. We train the SpaGCN with the recommended parameters, including s=1, b=49, p=0.5, and max_epochs=200, and set each one to generate the same number of clusters as the ground truth layers.

SEDR [Xu et al., 2024], the raw ST mRNA count data were preprocessed by the adata_preprocess function in the SEDR python package, and the position data were used to construct the graph using the graph_construction function at the same time. Then, we used the recommended parameters to train the SEDR network. Then, the Louvain clustering algorithm was used to identify clusters with latent embedding. Finally, we tried clustering at different resolutions to obtain the same number of clusters as the ground truth layers. We also used the K-means to cluster the latent embedding as the result of SEDR-K-means for

comparison.

STAGATE [Dong and Zhang, 2022], the raw ST mRNA count data and the spot positions were used as input. The count matrix input was read via the read_10X_Visium function in the STAGATE package. We sequentially used the highly_variable_genes, normalize_total and log1p functions to preprocess the data. Next, the spot positions were also used to construct the spatial network by using the Cal_Spatial_Net and Stats_Spatial_Net functions in the STAGATE package with the parameter setting of rad_cutoff=300. Then, we used train_STAGATE to train the network with the recommended parameters. Finally, latent embedding is used for domain definition via the mclust method. We also used the K-means to cluster the latent embedding as the result of STAGATE-K-means for comparison.

CCST [Li et al., 2022], the raw ST mRNA count data and the spot positions were used as input. The count matrix input was read via the Read_10X function in the CCST package. The adata_preprocess functions were used to preprocess data with the default parameter settings. Next, the spot positions were also used to construct the spatial network using the get_graph function in the CCST package. Then, we use the recommended parameters to train the DGI model. Finally, latent embedding is used for domain definition via the K-means method.

SpaceFlow [Ren et al., 2022], the raw ST mRNA count data and the spot positions were used as input. The count matrix input was read via the read_10X_Visium function in the SpaceFlow package. We sequentially used the filter_genes and preprocessing_data functions to preprocess data with the parameter settings of "min_cells=3" and "n_top_genes=3000". Next, we used the train function to train the network with the recommended parameters. Finally, the latent embedding is used for domain definition via the mclust method. Then, the Louvain clustering algorithm was used to identify clusters with latent embedding. Finally, we tried clustering at different resolutions to obtain the same number of clusters as the ground truth layers.

DeepST [Xu et al., 2022], the raw ST mRNA count data and the histological image of the tissue were used as input. The count matrix input was read and preprocessed via the get_adata and get_augment functions in the DeepST package, respectively. The histological image of the tissue was used to construct the graph by the get_graph function. At the same time, the preprocessed data were sequentially used as filter_genes with the parameter settings of "distType='BallTree'" and "k=12". Next, we used the fit function to train the network with the recommended parameters. Finally, latent embedding will then be used for domain definition via the get_cluster_data function using the Louvain clustering algorithm. We also used the K-means to cluster the latent embedding as the result of DeepST-K-means for comparison.

GraphST [Long et al., 2023], the raw ST mRNA count data and the spot positions were used as input. The count matrix and spatial coordinates were read using the *read_visium* function in the Scanpy [Wolf et al., 2018] package. GraphST was applied to learn low-dimensional latent embeddings by jointly modeling spatial proximity and gene expression profiles, using the default parameter settings recommended by the authors. After training, the learned latent embeddings were extracted for downstream analysis and spots without ground-truth annotations were excluded from subsequent analyses. The latent embeddings were then standardized and further reduced using principal component analysis (PCA) with 20 components. Finally, the reduced embeddings were clustered using the K-means algorithm, with the number of clusters set to the ground-truth number of tissue layers. The clustering performance was evaluated using the Adjusted Rand Index (ARI).

STMGAMF [Fu et al., 2025]. Genes expressed in fewer than 100 spots were filtered out, and highly variable genes were selected using the Seurat v3 method with the number of top genes fixed to 3000. The expression matrix was then normalized by library size to 10,000 counts per spot and scaled without zero-centering. Based on the preprocessed expression data, a feature graph was constructed using a k-nearest neighbor strategy in the gene expression space. In addition, a spatial graph was constructed according to the physical coordinates of spots, where spatial neighbors were defined within a fixed radius. Both the feature graph and spatial graph were used as inputs to STMGAMF for joint representation learning. Adaptive updating was enabled for both the feature graph and the spatial graph during training, while all other settings followed the default configuration provided by the authors. The model was trained using the Adam optimizer. After training,

the learned latent embeddings were extracted and used for downstream spatial domain identification, where K-means clustering was applied to obtain the final spatial domains.

4. Evaluation Metrics

For datasets with ground-truth annotations, we used the adjusted Rand index (ARI), Fowlkes–Mallows index (FMI) and normalized mutual information (NMI) to evaluate clustering performance. For the gene expression correction task, we used the Pearson correlation coefficient (PCC) to quantify the similarity between the recovered and experimentally measured gene expression values, and the correlation matrix difference (CMD) to evaluate whether the recovered expression matrix preserves the measured cell–cell similarity structure, thereby reflecting its ability to retain biological information.

ARI is used to calculate the similarity between the clustering labels obtained from a clustering algorithm and the reference cluster labels. Given a set of n cells and two sets of clustering labels of these cells, the overlap between the two sets of clustering labels can be summarized in a contingency table, in which each entry denotes the number of cells in common between the two sets of clustering labels. Specifically, the ARI is calculated as following:

$$\mathrm{ARI} = \frac{\sum_{jj'} \binom{n_{jj'}}{2} - \left[\sum_j \binom{a_j}{2} \sum_{j'} \binom{b_{j'}}{2} \Big/ \binom{n}{2}\right]}{\frac{1}{2}\left[\sum_j \binom{a_j}{2} + \sum_{j'} \binom{b_{j'}}{2}\right] - \left[\sum_j \binom{a_j}{2} \sum_{j'} \binom{b_{j'}}{2} \Big/ \binom{n}{2}\right]}$$

where $n_{jj'}$ is the number of cells assigned to cluster j based on the reference cluster labels and cluster j' based on clustering labels obtained from a clustering algorithm, a_j is the number of cells assigned to cluster j in the reference set, and $b_{j'}$ is the number of cells assigned to cluster j' by the clustering algorithm.

FMI is used to evaluate the similarity between the clustering labels obtained from a clustering algorithm and the reference cluster labels based on pairwise sample assignment. Given a set of n cells and two sets of clustering labels of these cells, the overlap between the two sets of clustering labels can be summarized in a contingency table. Specifically, the FMI is calculated as following:

$$\mathrm{FMI} = \frac{\sum_{jj'} \binom{n_{jj'}}{2}}{\sqrt{\left(\sum_j \binom{a_j}{2}\right)\left(\sum_{j'} \binom{b_{j'}}{2}\right)}}$$

where $n_{jj'}$ is the number of cells assigned to cluster j based on the reference cluster labels and cluster j' based on the clustering labels obtained from a clustering algorithm, a_j is the number of cells assigned to cluster j in the reference set, and $b_{j'}$ is the number of cells assigned to cluster j' by the clustering algorithm.

NMI leverages information theory to quantify the similarity between clusters. NMI ranges from 0 to 1, with higher values indicating better agreement between the clusters. In the following equation, N represents the number of samples, $I(\mathcal{C};\mathcal{G})$ denotes the mutual information between the predicted clusters $\mathcal{C}$ and the ground truth clusters $\mathcal{G}$, and $H(\mathcal{G})$ denotes the information entropy of the $\mathcal{G}$.

$$NMI = \frac{2 \cdot I(\mathcal{C};\mathcal{G})}{H(\mathcal{C}) + H(\mathcal{G})}$$

$$I(\mathcal{C};\mathcal{G}) = \sum_{d'} \sum_{d} P(V^{d'}, \mathcal{G}^d) \log \frac{P(\mathcal{C}^{d'}, \mathcal{G}^d)}{P(\mathcal{C}^{d'})P(\mathcal{G}^d)}$$

$$H(\mathcal{G}) = -\sum_{d} P(\mathcal{G}^d) \log P(\mathcal{G}^d)$$

$$P(\mathcal{G}^{d'}, \mathcal{C}^d) = \frac{|\mathcal{G}^{d'} \cap \mathcal{C}^d|}{N}, \quad P(\mathcal{G}^d) = \frac{|\mathcal{G}^d|}{N}$$

PCC is computed between the corrected and measured expression values for each gene. As defined in the follwing equation, x and y denote the measured and corrected gene expression vectors, respectively, $\bar{x}$ and $\bar{y}$ denote their means, and N denotes the number of spots.

$$\mathrm{PCC}(x, y) = \frac{\sum_{i=1}^{N}(x_i - \bar{x})(y_i - \bar{y})}{\sqrt{\sum_{i=1}^{N}(x_i - \bar{x})^2}\sqrt{\sum_{i=1}^{N}(y_i - \bar{y})^2}}$$

CMD provides a general measure of the discrepancy between two correlation matrices, R_1 and R_2, with smaller values indicating closer agreement. The CMD is defined as following, where trace$(\cdot)$ denotes the trace of a matrix and $\| \cdot \|_F$ represents the Frobenius norm.

$$\mathrm{CMD}(R_1, R_2) = 1 - \frac{\mathrm{trace}(R_1 R_2)}{\|R_1\|_F \|R_2\|_F}$$

5. GSG Parameter Sensitivity Analysis

We conducted a systematic evaluation of GSG hyperparameters on DLPFC dataset [Maynard et al., 2021]. First, we tested the effect of our graph-building method on the results by comparing it with K nearest neighbour (KNN) and shared nearest neighbour (SNN) on 12 DLPFCs and calculating the ARI values (Fig. S19A). Our graph-building method, which judges nearby spots using a circular region centered on the given spot, yielded much better ARI values than the other two methods. We also tested the impact of different circular radius and found that the best ARI values were obtained when there were fewer spot neighbouring nodes (Fig. S19B). Next, we tested the effect of feature selection using PCA and HVG methods (Fig. S19C). We found that feature selection was necessary for model construction and substantially improved model performance, with PCA being particularly well suited to our framework. We also tested the effect of different PCA downscaling dimensions (Fig. S19D) and different numbers of HVG-extracted genes (Fig. S19E) on the results and found that the different feature selection dimensions had a significant impact on the results, with PCA performing better than HVG for DLPFCs.

In addition, we explored the effects of different GNN architectures in the encoder and decoder. We found that using GIN symmetrically in both the encoder and decoder clearly outperformed the other tested configurations (Fig. S19E), and also achieved better performance than using an MLP as the decoder (Fig. S19F).

Finally, we performed a sensitivity analysis of the key GSG hyperparameter, masking ratio. According to Fig. S19G, the performance of GSG remained largely stable across masking ratios ranging from 0.2 to 0.9. Noticeable outliers only emerged when the masking ratio reached 0.9, suggesting that an excessively high masking ratio makes the reconstruction task overly difficult and leads to less stable model performance. In Fig. S20, we present the performance of GSG for each individual slice across different masking ratios, and GSG remained relatively stable on most slices, including 151508, 151510, 151669, and 151673. Overall, for datasets with relatively large and spatially continuous domains, such as DLPFC, we recommend the default masking ratio of 0.8. By contrast, for datasets with finer spatial structures, such as the mouse brain sections, overemphasizing spatial proximity may lead to over-smoothing. In these cases, a lower masking ratio may be more appropriate. For example, we used a masking ratio of 0.5 for the Visium mouse brain coronal dataset shown in Fig. S16B.

6. Computational Efficiency Analysis

To evaluate the computational efficiency of GSG, we conducted two complementary sets of experiments. First, we benchmarked the runtime of GSG against several baseline methods on a standard Visium dataset. Second, we utilized a large-scale dataset to systematically assess the impact of varying masking ratios on computational overhead. To ensure a rigorous and fair comparison, all experiments were conducted on a unified hardware platform using CPU execution only, specifically a server equipped with an Intel Core i7-5930K CPU @ 3.50GHz (12 logical processors) and 125 GB RAM.

For the initial benchmarking, we utilized the DLPFC dataset, comprising 12 tissue slices with approximately 3,000–5,000 spots each, providing a robust basis for runtime evaluation. The execution times for all methods across these slices are summarized as boxplots in Fig. S21A. The results demonstrate that while GSG is not the fastest among the compared methods, it remains highly computationally competitive, with model training for each DLPFC slice consistently completed within 120 seconds on the CPU.

We further evaluated the scalability of GSG using the large-scale Stereo-seq MOB dataset (19,527 cells) under masking ratios ranging from 0.2 to 0.9. As shown in Fig. S21B, C, even at a masking ratio of 0.9 (corresponding to 17,574 masked cells), the total runtime did not exceed 7 minutes.

This high efficiency is attributable to the architectural design of GSG. Under our masked prediction framework, message passing during forward propagation is performed on the original graph structure, thereby bypassing the computationally intensive data augmentation strategies typically required by contrastive learning methods. Additionally, the SCE loss computation is highly optimized, as it relies on efficient dot-product operations.Consequently, the total time complexity of GSG can be decomposed into four primary stages: graph masking, encoder propagation, decoder reconstruction, and SCE loss computation. The complexity is formally derived as follows, where p is the masking ratio, N is the number of nodes, E is the number of edges, d is the input feature dimension, h is the hidden dimension, and l_e and l_d denote the numbers of encoder and decoder layers, respectively:

$$\begin{gathered} T_{\text{epoch}} \approx T_{\text{mask}} + T_{\text{enc}} + T_{\text{remask}} + T_{\text{dec}} + T_{\text{loss}} \\ T_{\text{mask}} = T_{\text{loss}} = O(pNd), \qquad T_{\text{remask}} = O(pNh) \\ T_{\text{enc}} \approx O(l_e E h), \qquad T_{\text{dec}} \approx O(l_d E h) \\ T_{\text{epoch}} \approx O\big((l_e + l_d)Eh\big) + O\big(pN(d+h)\big) \end{gathered}$$

These results indicate that the time complexity of GSG is primarily dominated by the graph message-passing term within the encoding component, rendering the overall computational overhead largely invariant to changes in the masking ratio.

7. Data Availability

(1) Human DLPFCs within the spatialLIBD (http://spatial.libd.org/spatialLIBD);
(2) Human breast cancer and mouse brain tissue section datasets (https://support.10xgenomics.com/spatial-gene-expression/datasets);
(3) Stereo-seq dataset for mouse olfactory bulb tissue (https://github.com/BGIResearch/stereopy;
(4) 6F Slide-seqV2 data and seqFISH data (https://github.com/scverse/squidpy);
(5) fetal 9-13 week human heart single-cell RNA sequencing data (https://www.ncbi.nlm.nih.gov/geo/query/acc.cgi?acc=GSE10\protect\@normalcr\relax6118);
(6) human heart ST data of fetal 9 weeks (https://data.mendeley.com/datasets/dgnysc3zn5/1);
(7) Our in-house data: a healthy fetal human heart (9 weeks of gestation) of ST dataset has been deposited in Gene Expression Omnibus (GEO) with accession number GSE231496. Detailed descriptions of the sample collection and experimental procedures can be found in Supplementary Text 6.

8. Preprocessing of Internal Data

8.1 Human Sample Collection

Human heart tissues specimens were carefully sampled by a surgeon and immediately embedded using precooled OCT and stored in a -80°C refrigerator. The local ethics committee of The Obstetrics & Gynecology Hospital of Fudan University (Shanghai Red House Ob & Gyn Hospital) approved all human tissue protocols (No. 2024-106). The study met all relevant criteria of the code of conduct for responsible use of human tissue that is used in China. All patients provided informed consent, and the study was performed in accordance with the Declaration of Helsinki.

8.2 Human Heart Tissue Cryosection and Library Construction Using

Heart tissues were acclimated for 30 minutes in a -20°C freezing microtome before sectioning. RNA quality of the 10 µm cryosections was assessed using Agilent 2100 bioanalyzer. Continuous cryosectioned 10 µm to the largest section cutting plane was collected for ST sequencing (10x Genomics, Pleasanton, CA, USA).

After sectioning, tissue sections were adhered to Visium spatial gene expression slides (10x Genomics, 2000233), followed by fixation using precooled methanol (Sigma, 34860) for 30 min at -20°C and then stopped at 37°C for 1 min. Fixed tissue sections were subjected to H&E staining. The staining process was as follows: 7 min for Mayer's haematoxylin, 2 min for Dako bluing buffer, and 1 min for eosin Y. Slides were washed and incubated at 37°C for 5 min. Brightfield imaging was performed at 20X magnification with an inverted microscope (Olympus VS120).

Next, the tissue sections were permeabilized using a permeabilization enzyme in 0.1 M HCl buffer and incubated at 37°C for 30 min. Libraries were then constructed following the Visium Spatial Gene Expression User Guide (10x Genomics, CG000239). The RNA released from the tissues was reverse transcribed at 53°C for 45 minutes, and subsequently, the second strand was synthesized at 65°C for 15 minutes. The resulting cDNAs were enzymatically cleaved from the slides and amplified by PCR. The concentration of the PCR products was precisely quantified using a Qubit™ dsDNA Assay Kit (Thermo, Q32854). The cDNAs were then fragmented with Tn5 transposase at 32°C for 5 minutes, followed by ligation with dual index primers and PCR amplification. Finally, libraries were sequenced on an Illumina NextSeq PE150 sequencer with a depth of approximately 250 million reads for each sample, using 28 bp for read 1 and 120 bp for read 2.

8.3 Analysis of Human Heart ST Data from the Platform

Spatial RNA-seq data were trimmed and aligned to human transcriptome GRCh38 by running Space Ranger (v2.0). The dataset was processed using Seurat (v4.1.0). The counts were normalized with the SCTransform function and then processed in Seurat with a standard spatial RNA-seq pipeline. To identify cell–cell communication networks and regulons, we employed CellChat and performed SCENIC analysis as part of the standard pipeline [Aibar et al., 2017, Jin et al., 2021].

8.4 *In situ* Hybridization and Immunofluorescence

RNA-fluorescence *in situ* hybridization (FISH) experiments were performed as previously described with modifications. The expression of *CDH11* and *APCDD1* was evaluated by FISH [Pearson et al., 2009, King and Newmark, 2013]. Briefly, 10 µm cryosections were fixed for 10 min in 4% paraformaldehyde in PBS, followed by proteinase K treatment (1 µg/mL) for 5 min. After prehybridization for 1 hour, hybridization was performed at 56°C for over 16 hours, and the antibody was developed using the tyramide signal amplification system following extensive washes. Immunofluorescence was performed after all FISH steps.

To stain cardiomyocytes, a cardiac muscle troponin T (cTnT) antibody (1:200; Abcam, ab8295) was used with goat anti-mouse alexa647 secondary antibody (1:1000; Sigma, A21236) or goat anti-mouse cy3 secondary antibody (1:1000; Jackson ImmunoResearch, 115-165-146). To stain atrial cardiomyocytes, a myosin regulatory light chain 2, atrial isoform (MLC-2a) antibody (1:200; Synaptic Systems, 311011) was used with goat anti-mouse cy3 secondary antibody (1:1000; Jackson ImmunoResearch, 115-165-146). To stain Reelin protein in the heart, a Reelin antibody (1:200; HuaBio, ET1704-98) was used with goat anti-rabbit alexa647 secondary antibody (1:1000; Jackson ImmunoResearch, A21245).

Fluorescent images were captured using an Olympus SpinSR disk and were processed using ImageJ.

Supplementary Tables

Supplementary Table S1. Model architecture and training parameters used in GSG.

Dataset	Platform	Layers	Hidden dimensions	Activation	Epoch	Optimizer	lr	weight_decay
DLPFC	Visium	3	128	ELU	500	Adam	0.001	2e-4
HBRC	Visium	3	128	ELU	500	Adam	0.001	2e-4
Mouse brain	Visium	2	128	ELU	500	Adam	0.001	2e-4
Mouse olfactory bulb	Stereo-seq	3	128	ELU	500	Adam	0.001	2e-4
Mouse hippocampus	Slide-seqV2	3	128	PReLU	850	Adam	0.001	2e-4
Mouse embryo	seqFISH	3	128	ELU	500	Adam	0.001	2e-4

Supplementary Figures

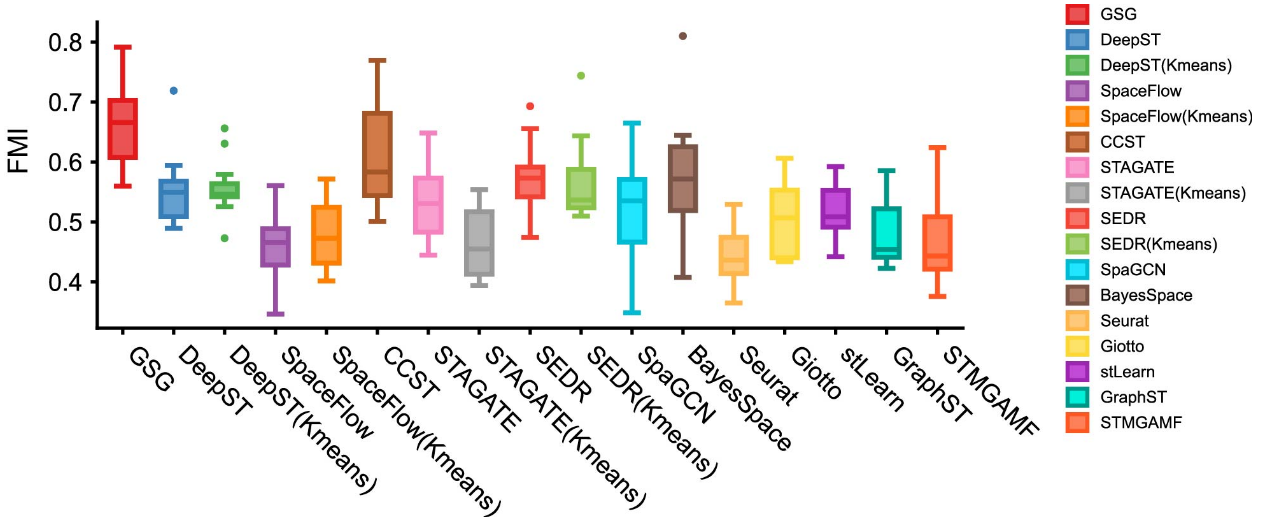

Supplementary Figure 1. Benchmarking GSG and baseline models on DLPFC dataset using FMI. Boxplot of FMI scores of GSG and 12 comparison methods across 12 slices. The center line, box limits, and whiskers represent the median, upper, and lower quartiles, and 1.5× interquartile range, respectively.

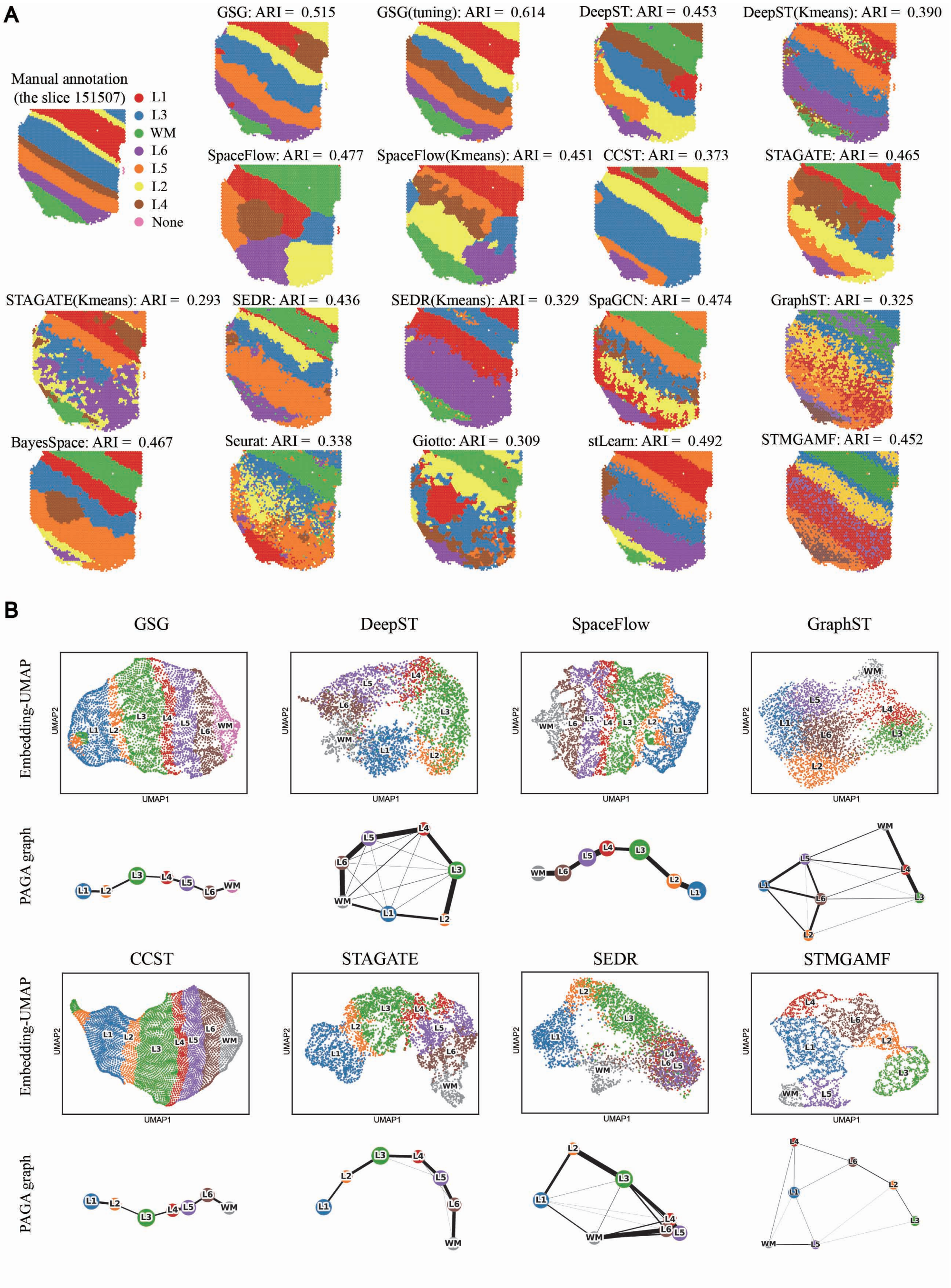

Supplementary Figure 2. Comparison between GSG and other methods for domains identification on the slice 151507 of human dorsolateral prefrontal cortex data. A. Ground-truth segmentation of cortical layers (L1 -L6) and white matter (WM) in the DLPFC section and Cluster assignments generated by 18 methods, GSG achieves the highest. **B.** UMAP and PAGA graphs generated by GSG, DeepST, SpaceFlow, CCST, STAGATE, SEDR, GraphST and STMGAMF embeddings.

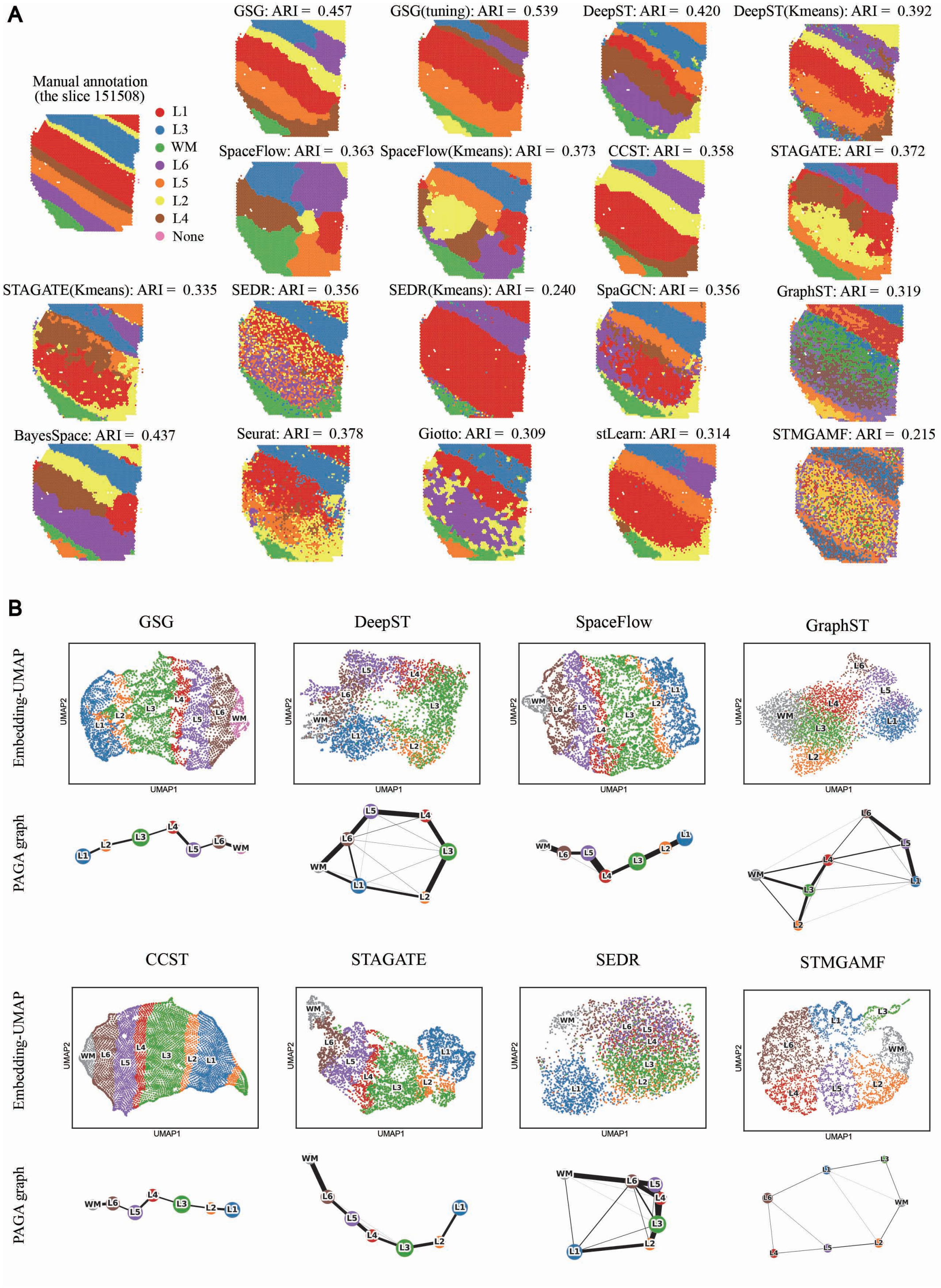

Supplementary Figure 3. Comparison between GSG and other methods for domains identification on the slice 151508 of human dorsolateral prefrontal cortex data. A. Ground-truth segmentation of cortical layers (L1 -L6) and white matter (WM) in the DLPFC section and Cluster assignments generated by 18 methods, GSG achieves the highest. **B.** UMAP and PAGA graphs generated by GSG, DeepST, SpaceFlow, CCST, STAGATE, SEDR, GraphST and STMGAMF embeddings.

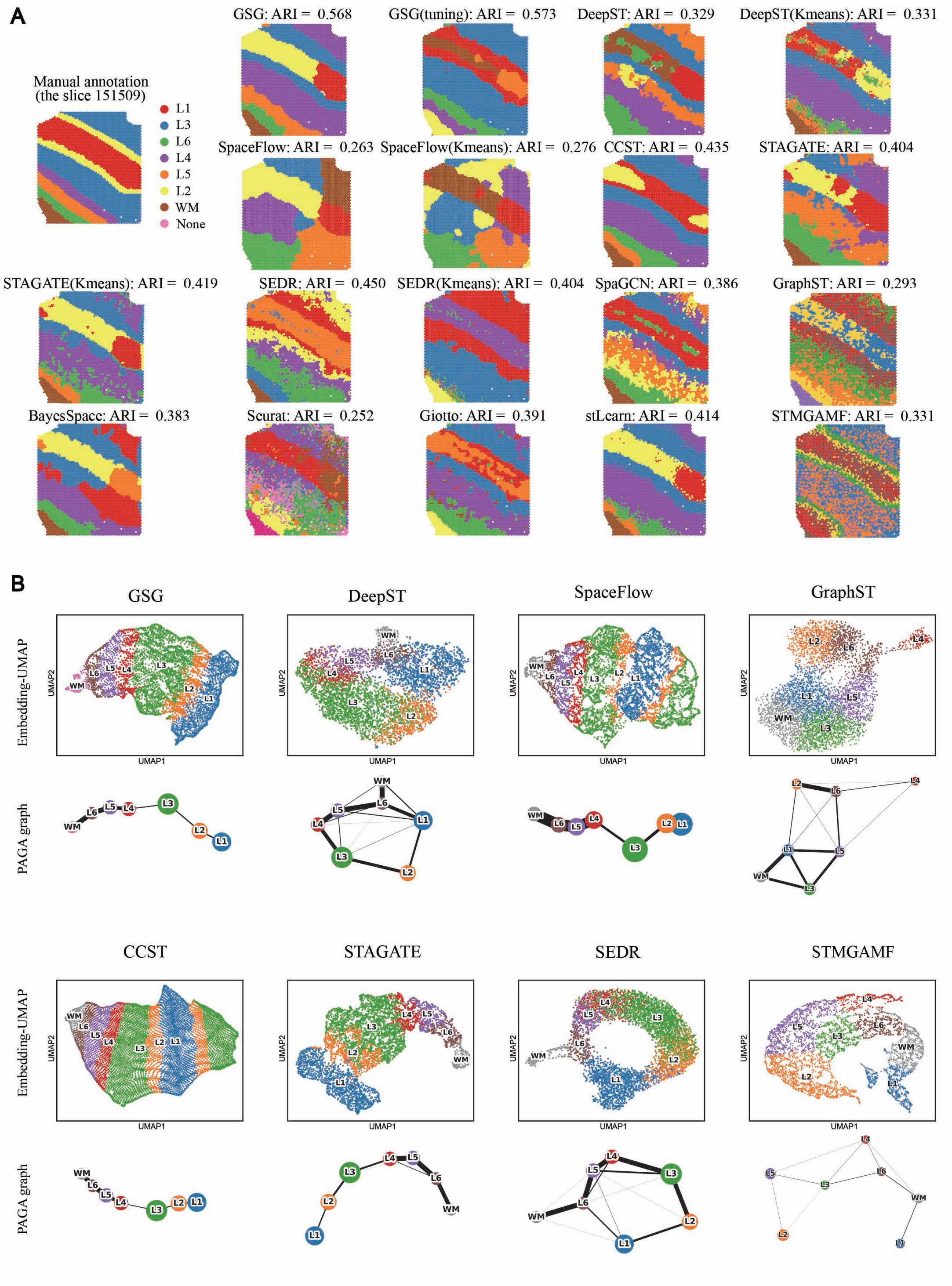

Supplementary Figure 4. Comparison between GSG and other methods for domains identification on the slice 151509 of human dorsolateral prefrontal cortex data. A. Ground-truth segmentation of cortical layers (L1 -L6) and white matter (WM) in the DLPFC section and Cluster assignments generated by 18 methods, GSG achieves the highest. **B.** UMAP and PAGA graphs generated by GSG, DeepST, SpaceFlow, CCST, STAGATE, SEDR, GraphST and STMGAMF embeddings.

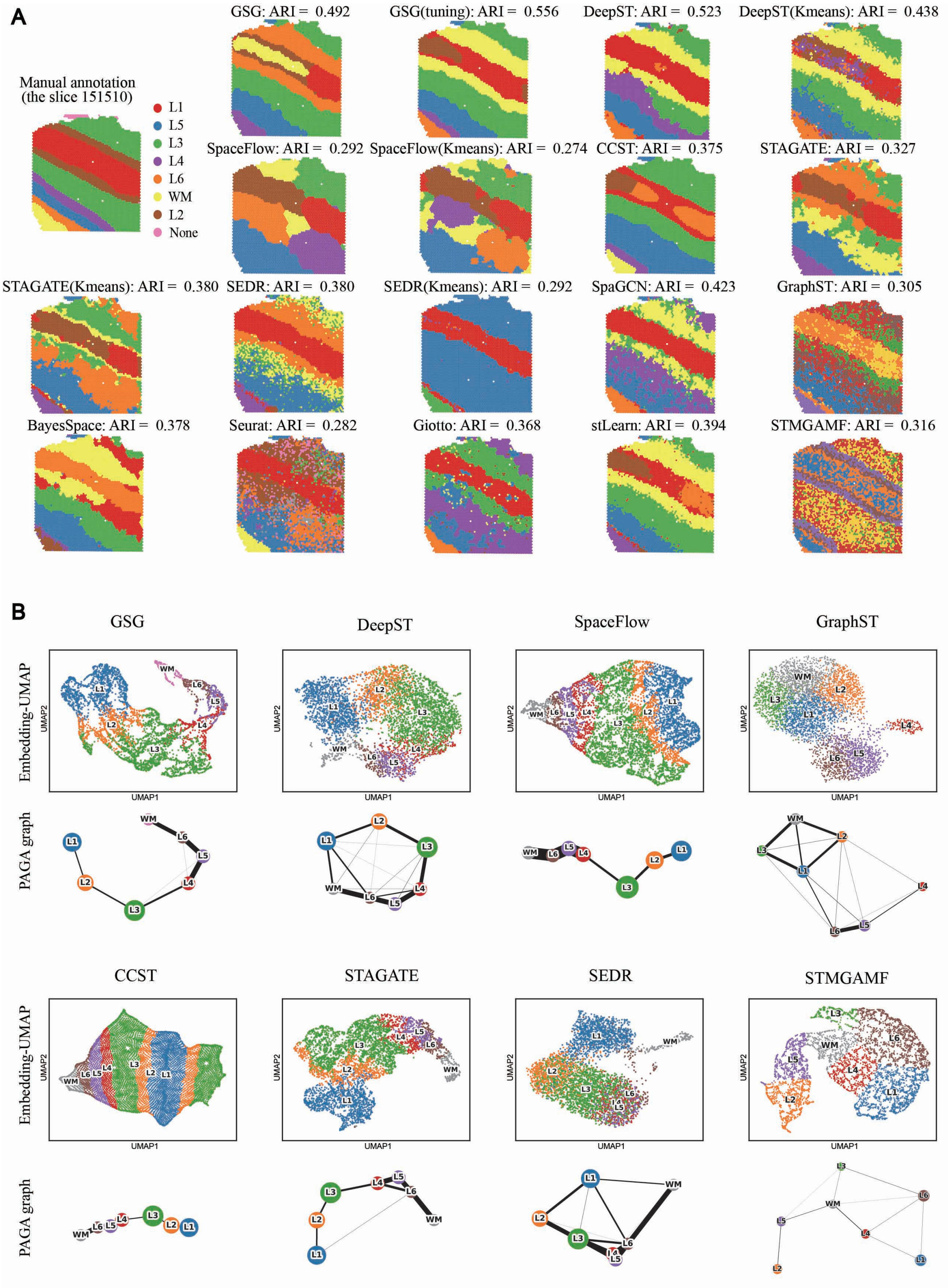

Supplementary Figure 5. Comparison between GSG and other methods for domains identification on the slice 151510 of human dorsolateral prefrontal cortex data. A. Ground-truth segmentation of cortical layers (L1 -L6) and white matter (WM) in the DLPFC section and Cluster assignments generated by 18 methods, GSG achieves the highest. **B.** UMAP and PAGA graphs generated by GSG, DeepST, SpaceFlow, CCST, STAGATE, SEDR, GraphST and STMGAMF embeddings.

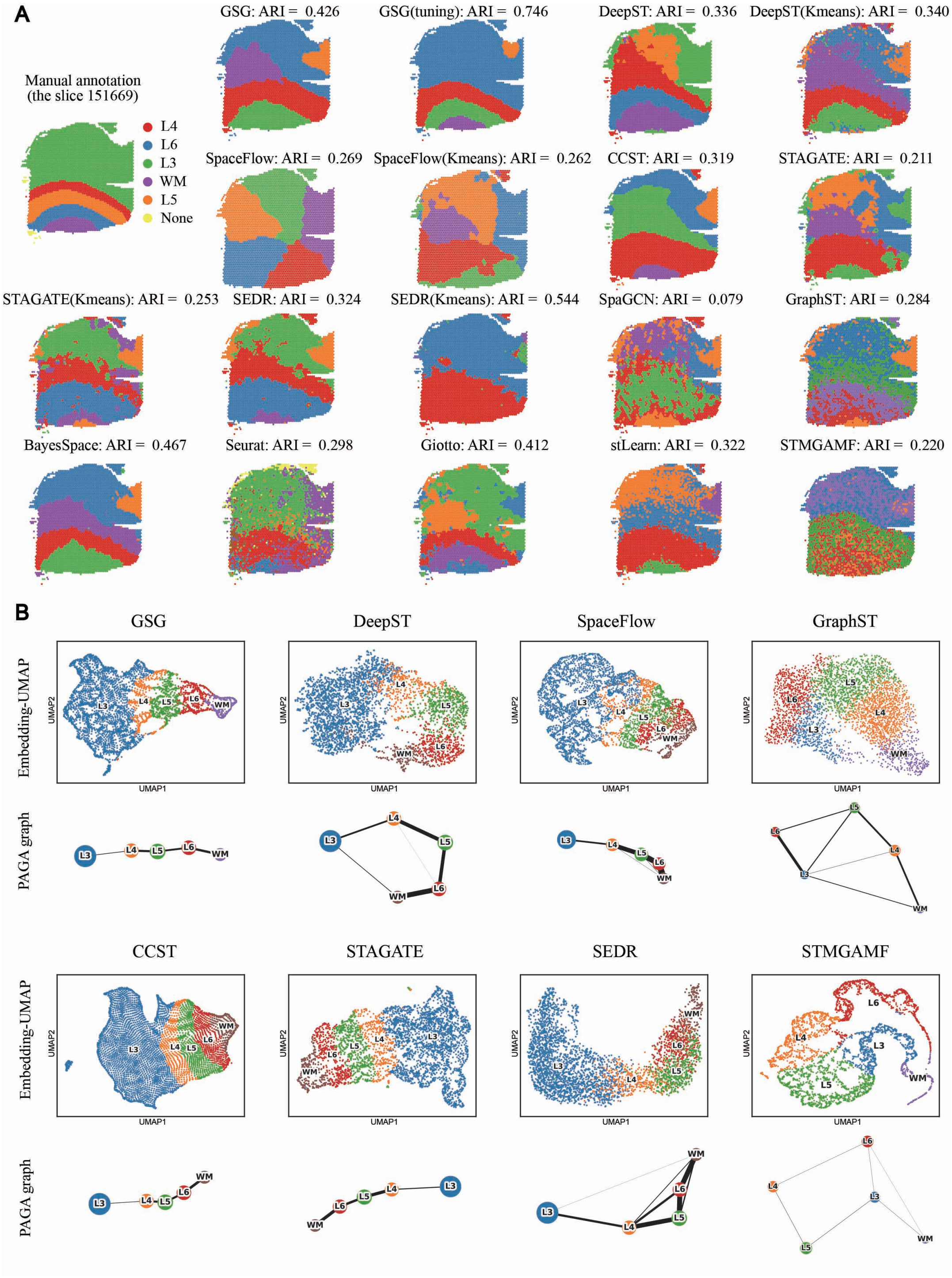

Supplementary Figure 6. Comparison between GSG and other methods for domains identification on the slice 151669 of human dorsolateral prefrontal cortex data. A. Ground-truth segmentation of cortical layers (L3 -L6) and white matter (WM) in the DLPFC section and Cluster assignments generated by 18 methods, GSG achieves the highest. **B.** UMAP and PAGA graphs generated by GSG, DeepST, SpaceFlow, CCST, STAGATE, SEDR, GraphST and STMGAMF embeddings.

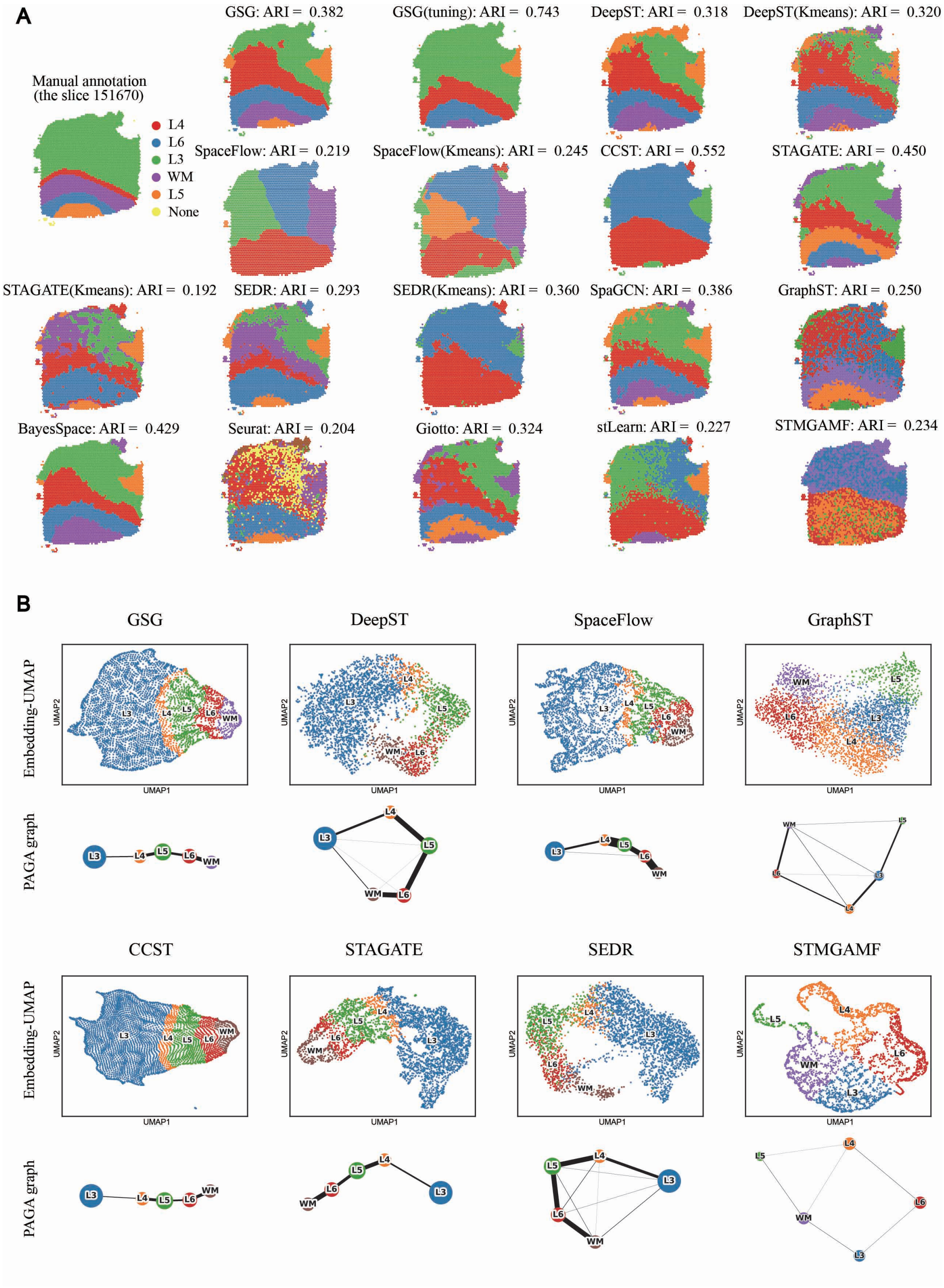

Supplementary Figure 7. Comparison between GSG and other methods for domains identification on the slice 151670 of human dorsolateral prefrontal cortex data. A. Ground-truth segmentation of cortical layers (L3 -L6) and white matter (WM) in the DLPFC section and Cluster assignments generated by 18 methods, GSG achieves the highest. **B.** UMAP and PAGA graphs generated by GSG, DeepST, SpaceFlow, CCST, STAGATE, SEDR, GraphST and STMGAMF embeddings.

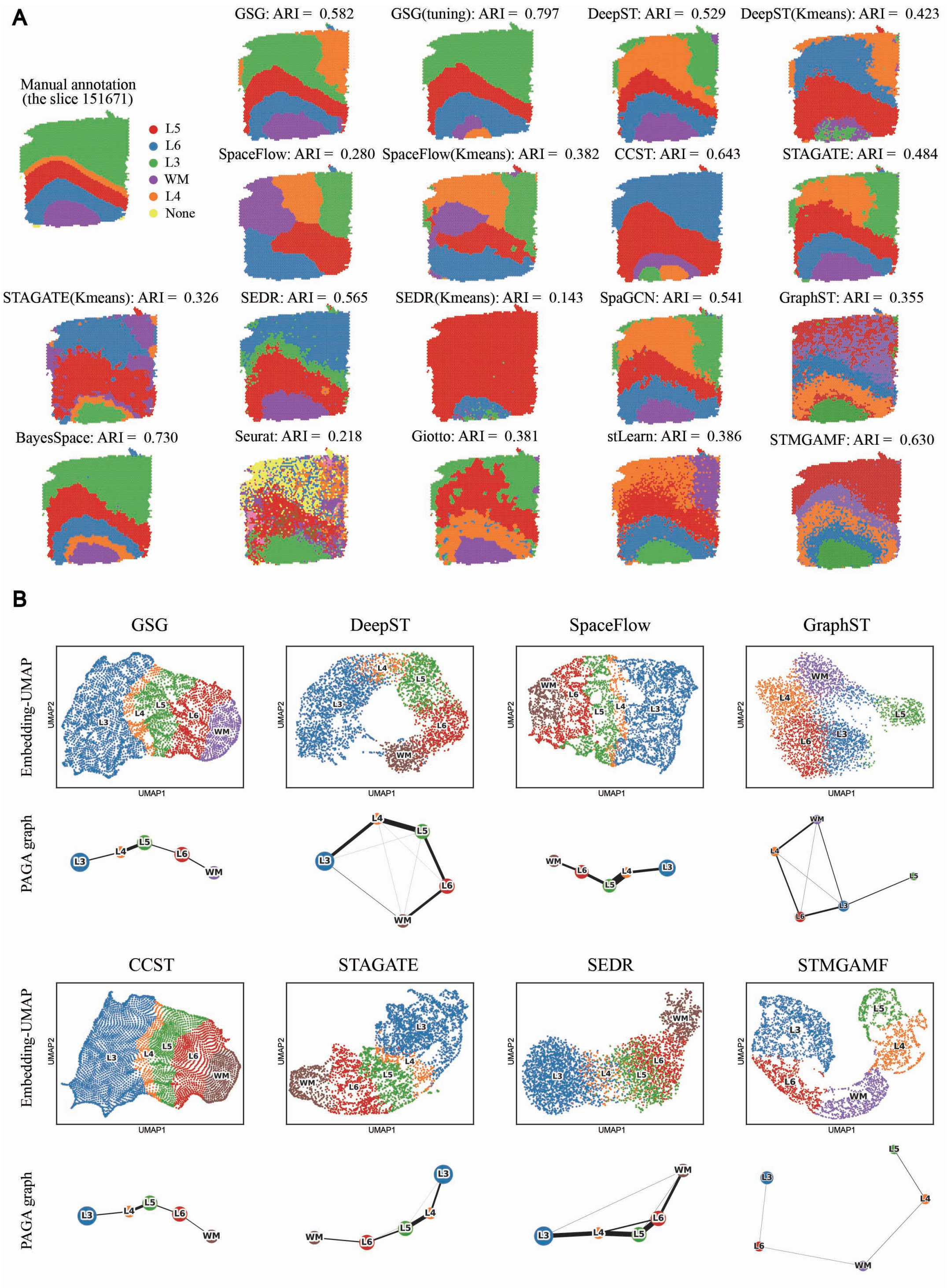

Supplementary Figure 8. Comparison between GSG and other methods for domains identification on the slice 151671 of human dorsolateral prefrontal cortex data. A. Ground-truth segmentation of cortical layers (L3 -L6) and white matter (WM) in the DLPFC section and Cluster assignments generated by 18 methods, GSG achieves the highest. **B.** UMAP and PAGA graphs generated by GSG, DeepST, SpaceFlow, CCST, STAGATE, SEDR, GraphST and STMGAMF embeddings.

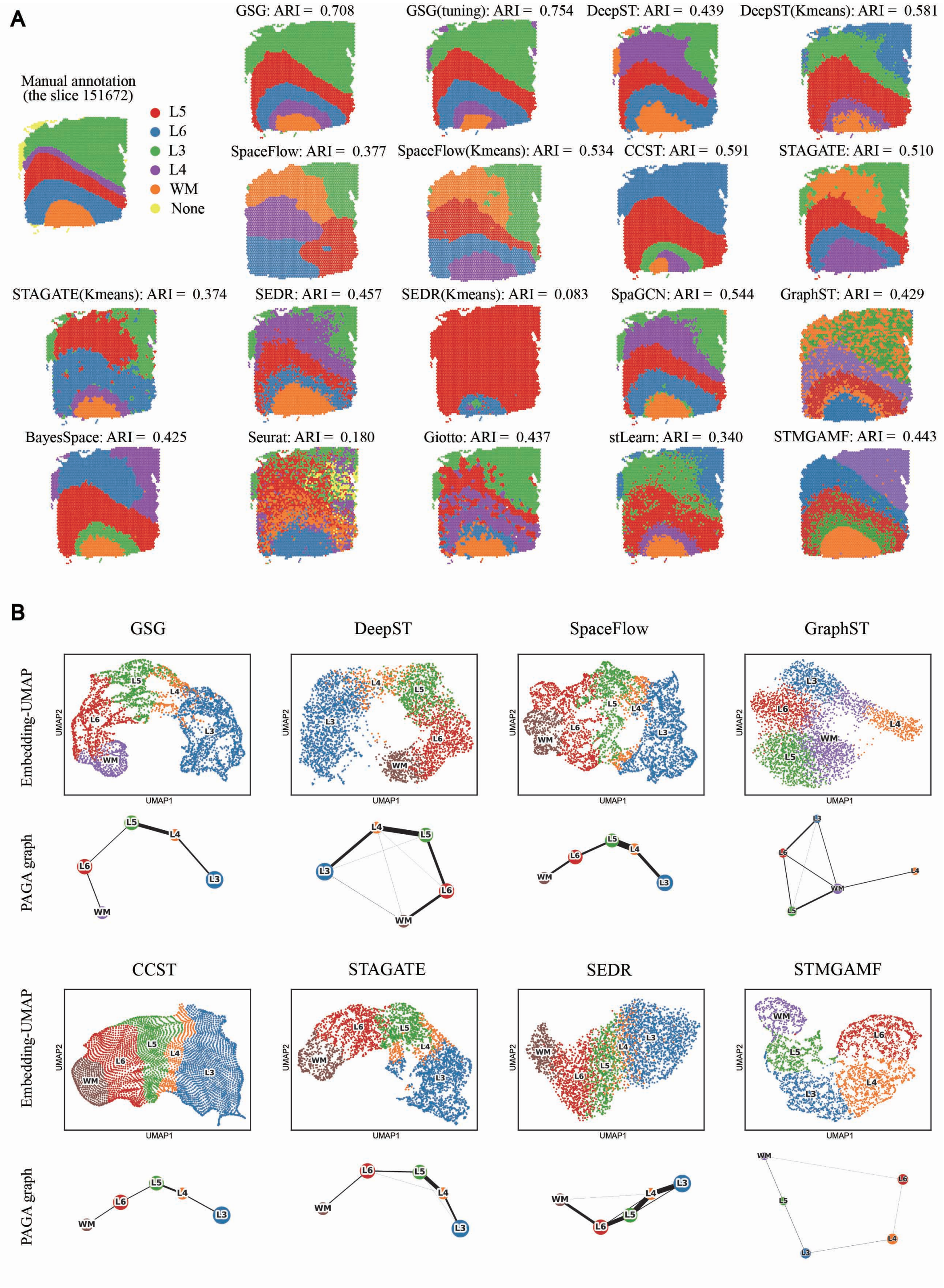

Supplementary Figure 9. Comparison between GSG and other methods for domains identification on the slice 151672 of human dorsolateral prefrontal cortex data. A. Ground-truth segmentation of cortical layers (L3 -L6) and white matter (WM) in the DLPFC section and Cluster assignments generated by 18 methods, GSG achieves the highest. **B.** UMAP and PAGA graphs generated by GSG, DeepST, SpaceFlow, CCST, STAGATE, SEDR, GraphST and STMGAMF embeddings.

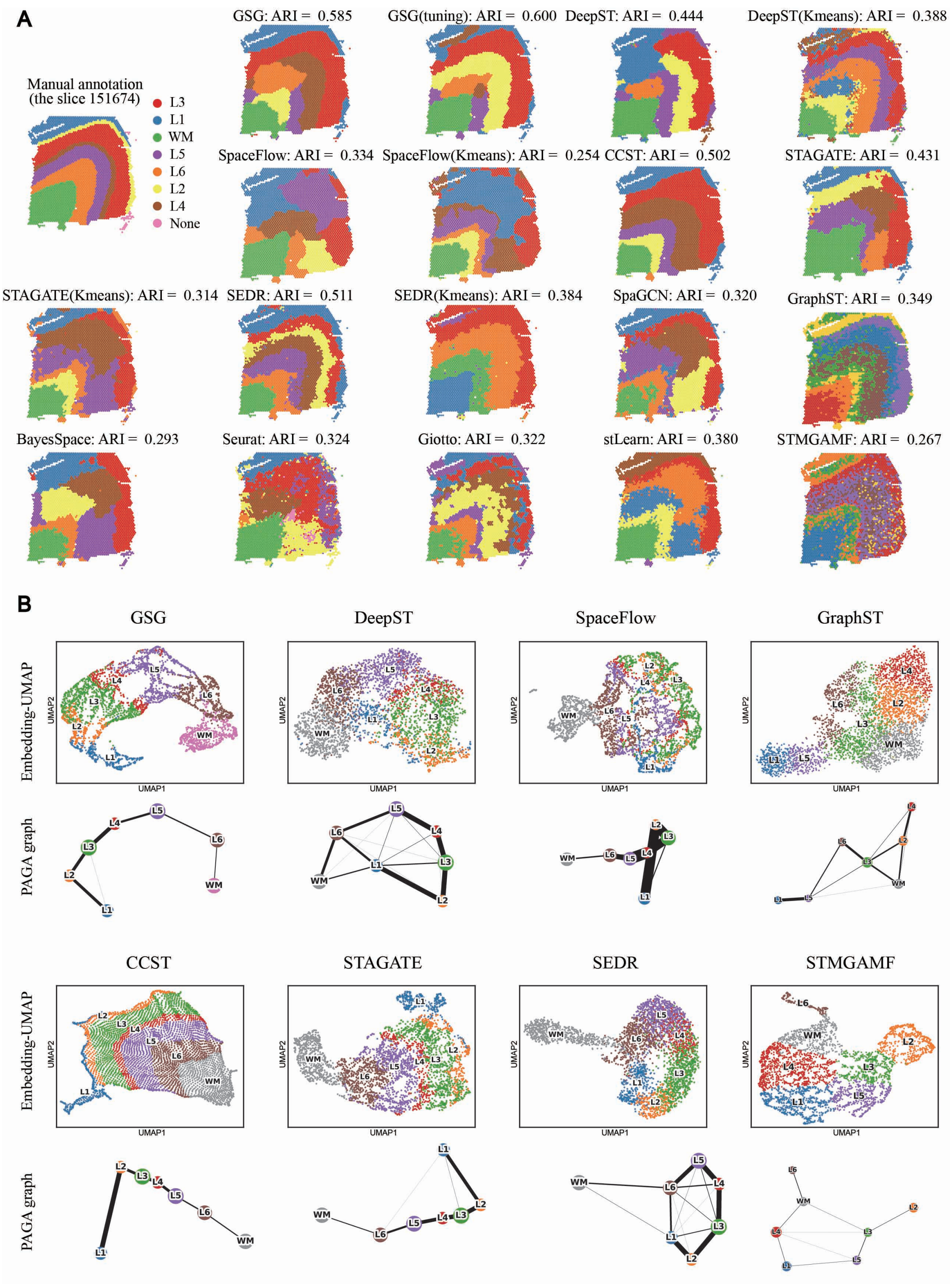

Supplementary Figure 10. Comparison between GSG and other methods for domains identification on the slice 151674 of human dorsolateral prefrontal cortex data. A. Ground-truth segmentation of cortical layers (L1 -L6) and white matter (WM) in the DLPFC section and Cluster assignments generated by 18 methods, GSG achieves the highest. **B.** UMAP and PAGA graphs generated by GSG, DeepST, SpaceFlow, CCST, STAGATE, SEDR, GraphST and STMGAMF embeddings.

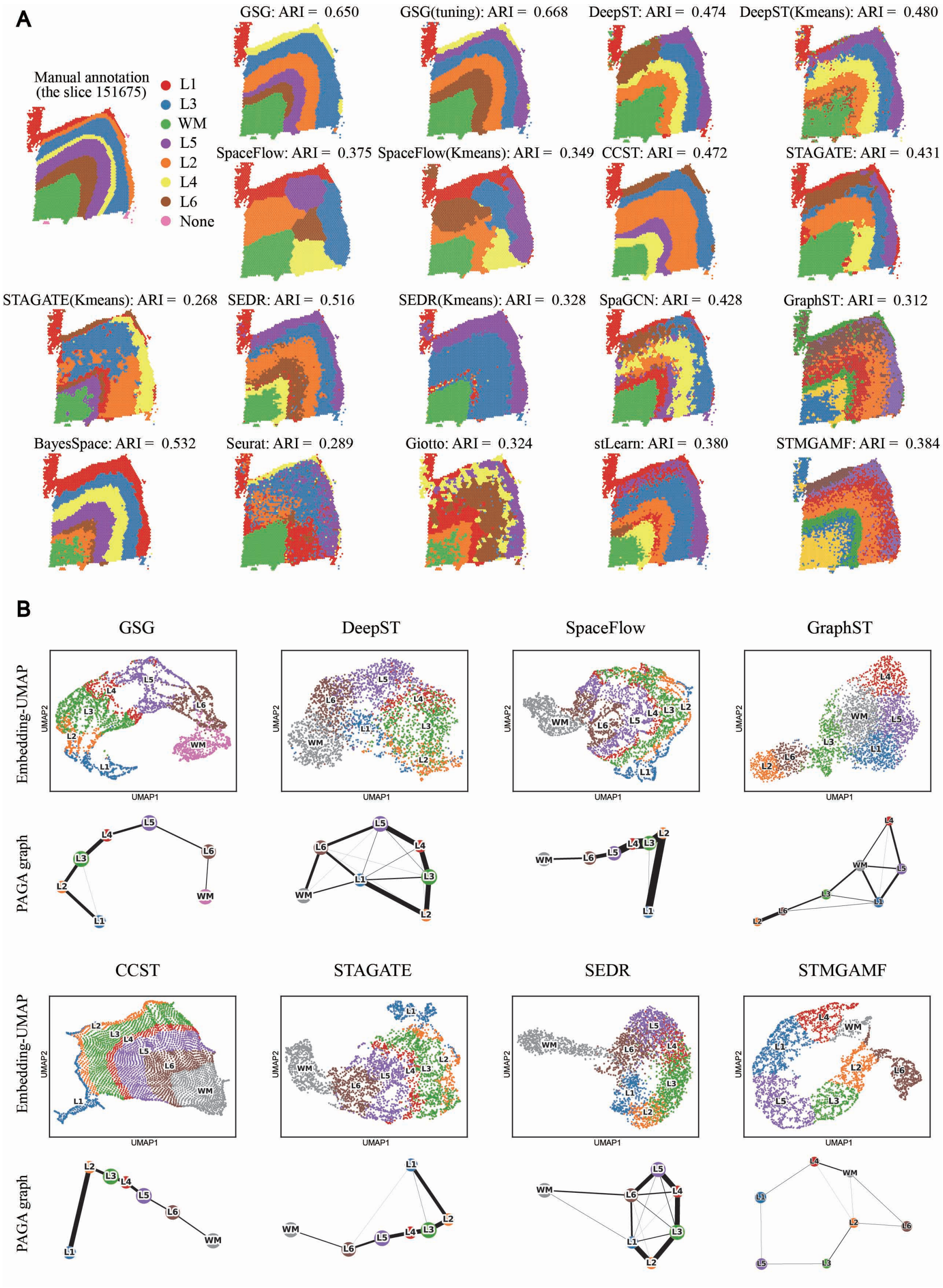

Supplementary Figure 11. Comparison between GSG and other methods for domains identification on the slice 151675 of human dorsolateral prefrontal cortex data. A. Ground-truth segmentation of cortical layers (L1 -L6) and white matter (WM) in the DLPFC section and Cluster assignments generated by 18 methods, GSG achieves the highest. **B.** UMAP and PAGA graphs generated by GSG, DeepST, SpaceFlow, CCST, STAGATE, SEDR, GraphST and STMGAMF embeddings.

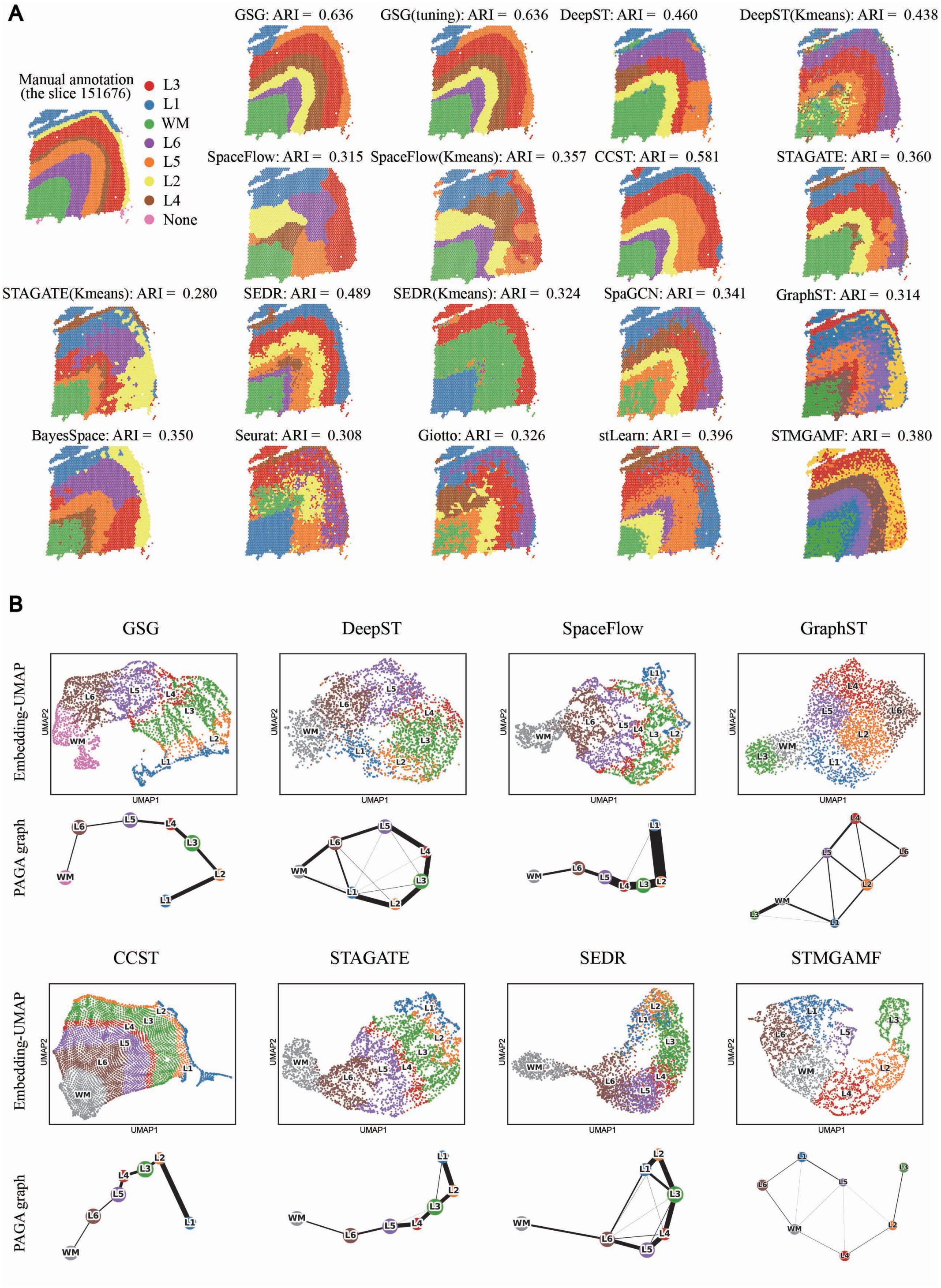

Supplementary Figure 12. Comparison between GSG and other methods for domains identification on the slice 151676 of human dorsolateral prefrontal cortex data. A. Ground-truth segmentation of cortical layers (L1 -L6) and white matter (WM) in the DLPFC section and Cluster assignments generated by 18 methods, GSG achieves the highest. **B.** UMAP and PAGA graphs generated by GSG, DeepST, SpaceFlow, CCST, STAGATE, SEDR, GraphST and STMGAMF embeddings.

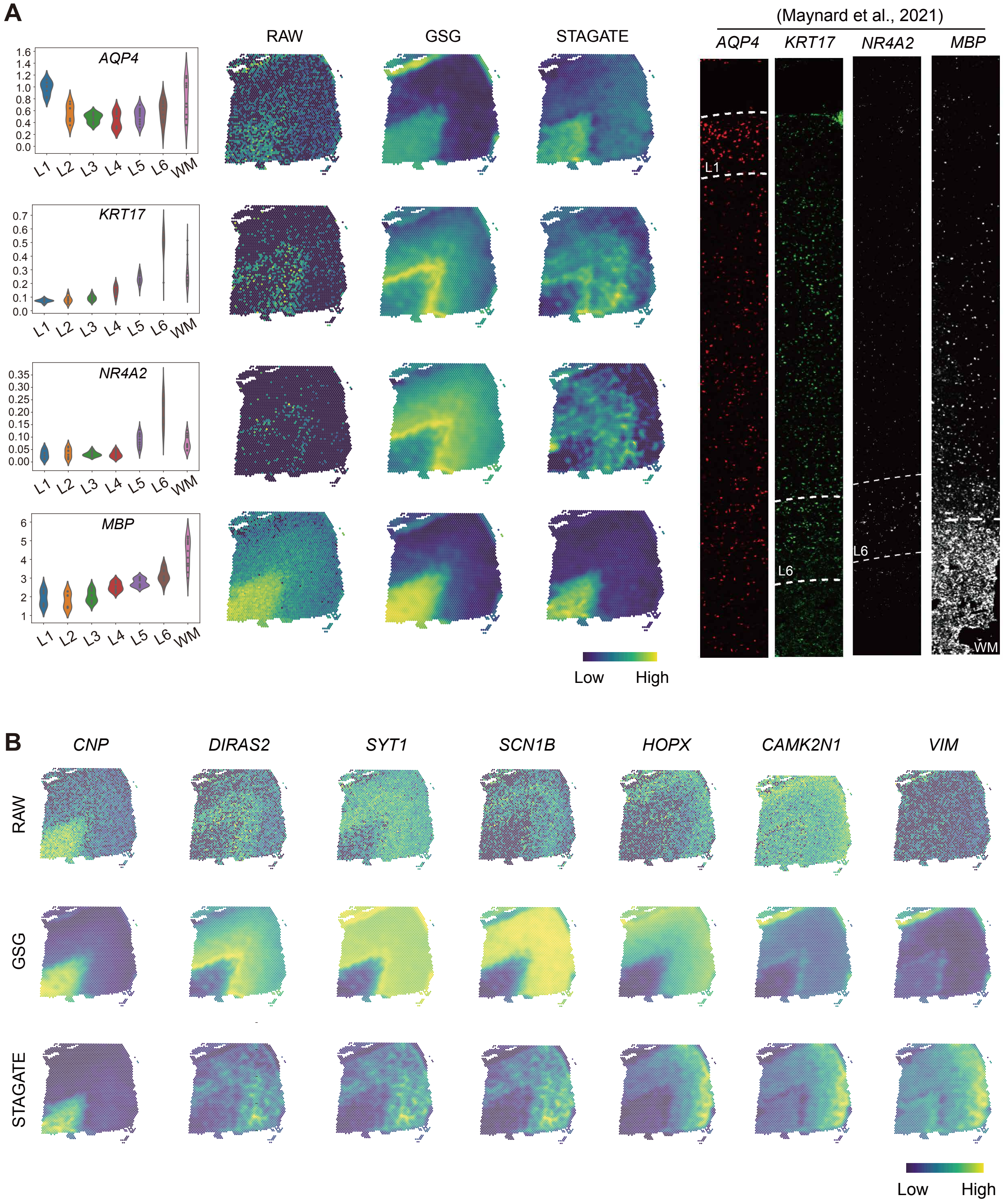

Supplementary Figure 13. Representative results of gene correction. Spatial visualizations of raw expressions (top), GSG gene correction (middle) and STAGATE gene correction (bottom). The gene order (from left to right) is consistent with the intensity of expression at spatial locations (from the inside out).

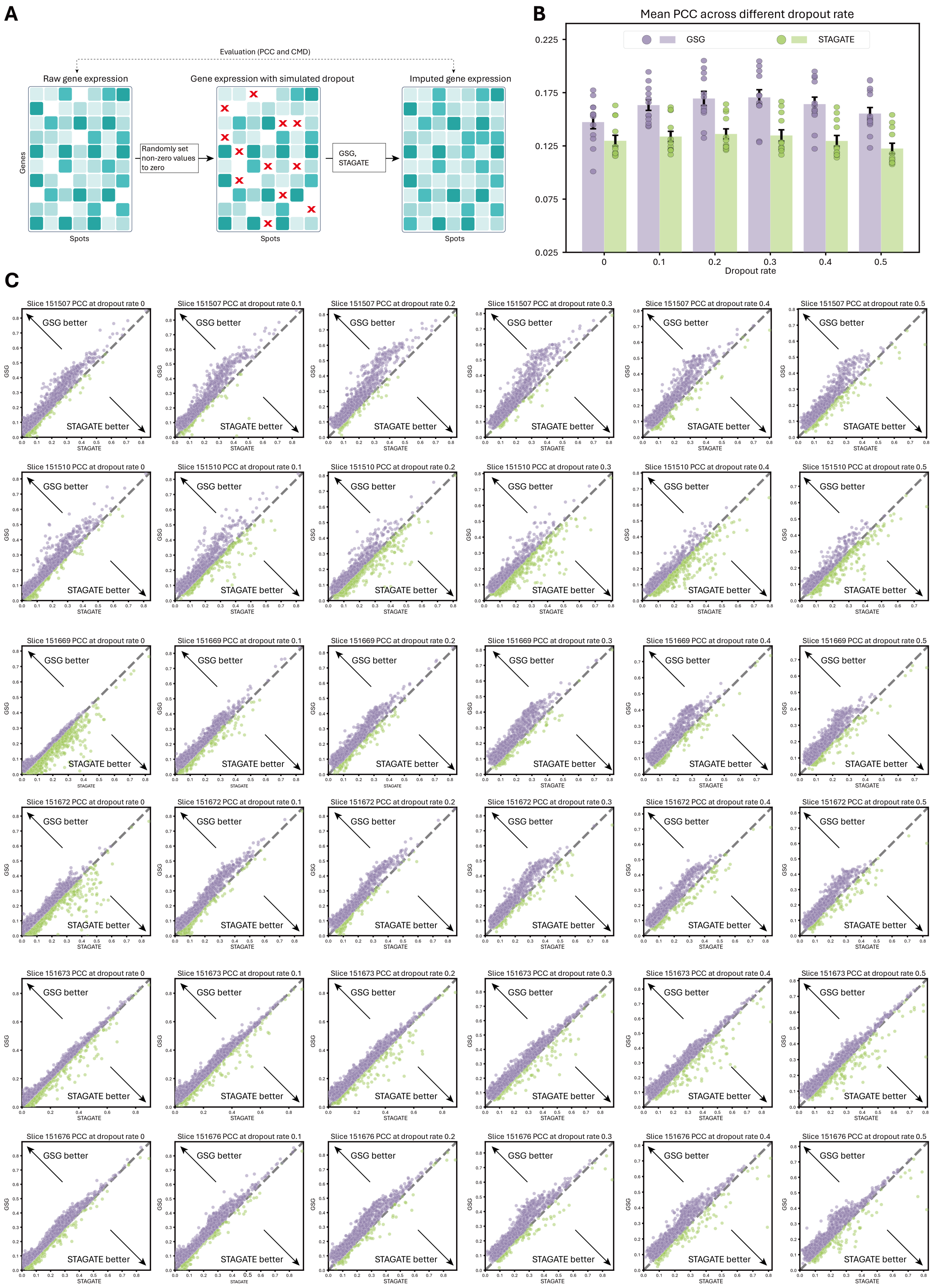
A
Evaluation (PCC and CMD)
Raw gene expression
Gene expression with simulated dropout
Imputed gene expression
Genes
Spots
Randomly set non-zero values to zero
GSG, STAGATE
B
Mean PCC across different dropout rate
GSG
STAGATE
Dropout rate
C
Slice 151507 PCC at dropout rate 0
Slice 151507 PCC at dropout rate 0.1
Slice 151507 PCC at dropout rate 0.2
Slice 151507 PCC at dropout rate 0.3
Slice 151507 PCC at dropout rate 0.4
Slice 151507 PCC at dropout rate 0.5
Slice 151510 PCC at dropout rate 0
Slice 151510 PCC at dropout rate 0.1
Slice 151510 PCC at dropout rate 0.2
Slice 151510 PCC at dropout rate 0.3
Slice 151510 PCC at dropout rate 0.4
Slice 151510 PCC at dropout rate 0.5
Slice 151669 PCC at dropout rate 0
Slice 151669 PCC at dropout rate 0.1
Slice 151669 PCC at dropout rate 0.2
Slice 151669 PCC at dropout rate 0.3
Slice 151669 PCC at dropout rate 0.4
Slice 151669 PCC at dropout rate 0.5
Slice 151672 PCC at dropout rate 0
Slice 151672 PCC at dropout rate 0.1
Slice 151672 PCC at dropout rate 0.2
Slice 151672 PCC at dropout rate 0.3
Slice 151672 PCC at dropout rate 0.4
Slice 151672 PCC at dropout rate 0.5
Slice 151673 PCC at dropout rate 0
Slice 151673 PCC at dropout rate 0.1
Slice 151673 PCC at dropout rate 0.2
Slice 151673 PCC at dropout rate 0.3
Slice 151673 PCC at dropout rate 0.4
Slice 151673 PCC at dropout rate 0.5
Slice 151676 PCC at dropout rate 0
Slice 151676 PCC at dropout rate 0.1
Slice 151676 PCC at dropout rate 0.2
Slice 151676 PCC at dropout rate 0.3
Slice 151676 PCC at dropout rate 0.4
Slice 151676 PCC at dropout rate 0.5
GSG better
STAGATE better
GSG
STAGATE

Supplementary Figure 14. Quantitative evaluation of the gene correction task on the DLPFC dataset. **A.** Schematic overview of the evaluation pipeline. Non-zero entries in the original sequencing output were randomly masked to zero at predefined dropout ratios to generate simulated dropout data. The corrupted data were then used as input to GSG and STAGATE, and the imputed gene expression values were quantitatively compared with the original expression values before dropout simulation. Performance was evaluated using PCC and CMD. **B.** Bar plot of the mean PCC across genes for 12 DLPFC slices under different dropout ratios. Bar heights indicate the mean of the slice-level mean PCC values across the 12 slices. Error bars represent the standard error of the mean (SEM). Each dot represents the mean PCC across genes for one slice (n=12). **C.** Scatter plots showing the PCC values of individual genes for the imputed expression generated by GSG and STAGATE across six representative DLPFC slices.

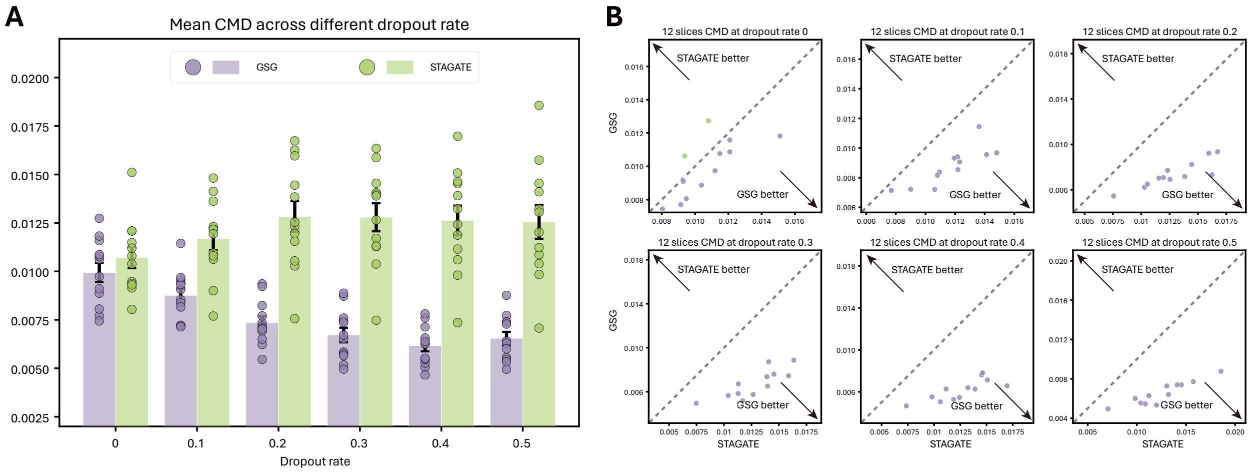

Supplementary Figure 15. Quantitative evaluation of the gene correction task on the DLPFC dataset using CMD. A. Bar plot of the mean CMD for 12 DLPFC slices under different dropout ratios. Bar heights indicate the mean of the slice-level mean CMD values across the 12 slices. Error bars represent the SEM. Each dot represents the mean CMD of one slice (n=12). **B.** Scatter plots showing the CMD values of the imputed expression generated by GSG and STAGATE across 12 DLPFC slices. Lower CMD indicates better correction performance.

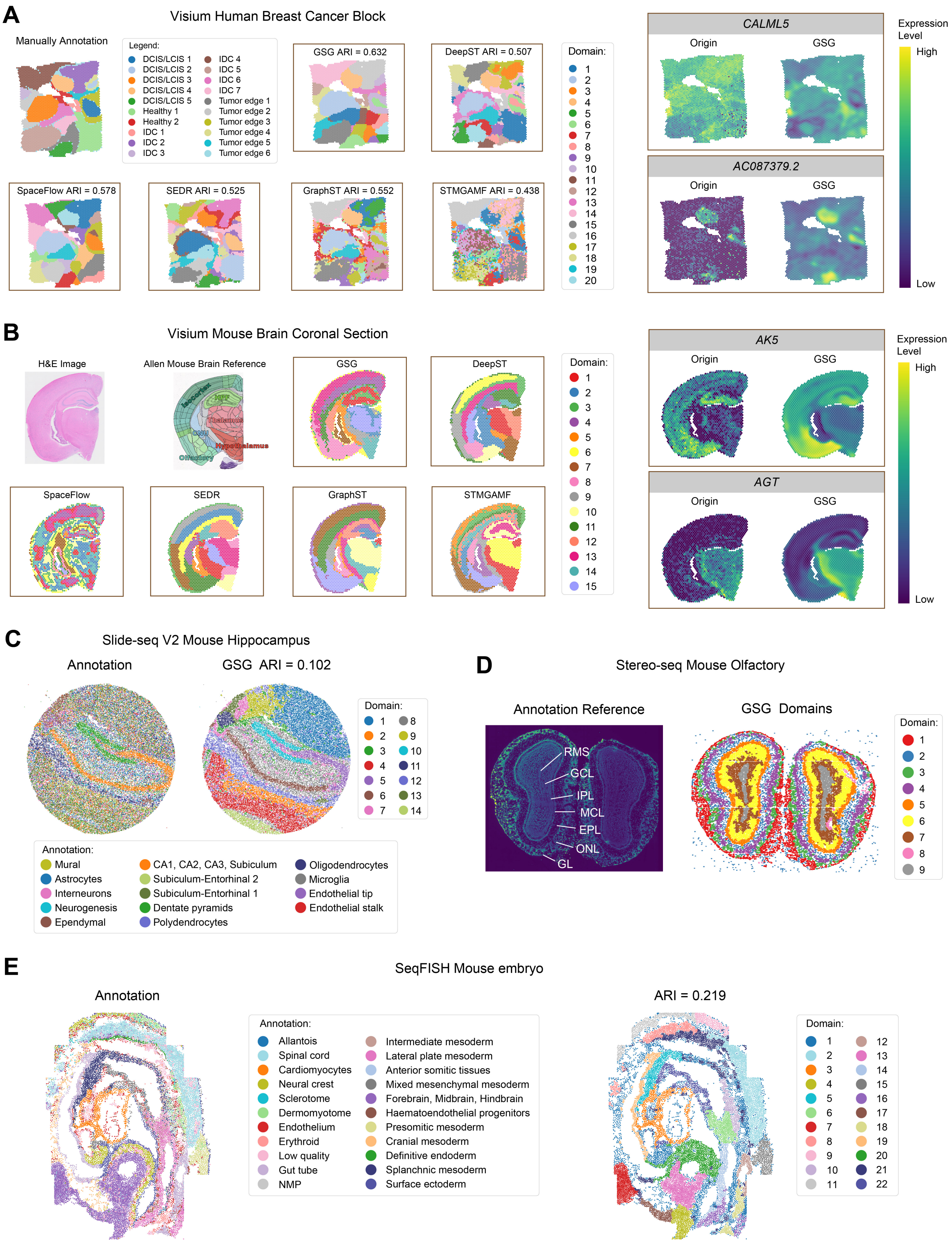
A
Visium Human Breast Cancer Block
Manually Annotation
Legend:
DCIS/LCIS 1
DCIS/LCIS 2
DCIS/LCIS 3
DCIS/LCIS 4
DCIS/LCIS 5
Healthy 1
Healthy 2
IDC 1
IDC 2
IDC 3
IDC 4
IDC 5
IDC 6
IDC 7
Tumor edge 1
Tumor edge 2
Tumor edge 3
Tumor edge 4
Tumor edge 5
Tumor edge 6
GSG ARI = 0.632
DeepST ARI = 0.507
SpaceFlow ARI = 0.578
SEDR ARI = 0.525
GraphST ARI = 0.552
STMGAMF ARI = 0.438
Domain:
CALML5
Origin
GSG
AC087379.2
Expression Level
High
Low
B
Visium Mouse Brain Coronal Section
H&E Image
Allen Mouse Brain Reference
GSG
DeepST
SpaceFlow
SEDR
GraphST
STMGAMF
AK5
AGT
C
Slide-seq V2 Mouse Hippocampus
Annotation
GSG ARI = 0.102
Annotation:
Mural
Astrocytes
Interneurons
Neurogenesis
Ependymal
CA1, CA2, CA3, Subiculum
Subiculum-Entorhinal 2
Subiculum-Entorhinal 1
Dentate pyramids
Polydendrocytes
Oligodendrocytes
Microglia
Endothelial tip
Endothelial stalk
D
Stereo-seq Mouse Olfactory
Annotation Reference
RMS
GCL
IPL
MCL
EPL
ONL
GL
GSG Domains
E
SeqFISH Mouse embryo
Annotation
ARI = 0.219
Allantois
Spinal cord
Cardiomyocytes
Neural crest
Sclerotome
Dermomyotome
Endothelium
Erythroid
Low quality
Gut tube
NMP
Intermediate mesoderm
Lateral plate mesoderm
Anterior somitic tissues
Mixed mesenchymal mesoderm
Forebrain, Midbrain, Hindbrain
Haematoendothelial progenitors
Presomitic mesoderm
Cranial mesoderm
Definitive endoderm
Splanchnic mesoderm
Surface ectoderm

Supplementary Figure 16. GSG generalizes well across tissue types and ST platforms. A. Left: Annotation on Visium human breast cancer section; spatial domains generated by GSG, DeepST, SpaceFlow, SEDR, GraphST and STMGAMF. Right: The gene corrected result by GSG. **B.** Left: H&E image of Visium coronal mouse brain section; anatomical reference from Allen Mouse Brain Atlas (`https://atlas.brain-map.org/`); spatial domains generated by GSG, DeepST, SpaceFlow, SEDR, GraphST and STMGAMF. Right: Gene correction result by GSG. HPF: hippocampal formation, CNU: cerebral nuclei. **C.** The annotation and GSG-identified spatial domain on Stereo-seq mouse olfactory bulb. **D.** The result of Slide-seqV2 data of mouse hippocampus spatial domain identification. **E.** The annotation and GSG-identified spatial domains on seqFISH mouse embryo section.

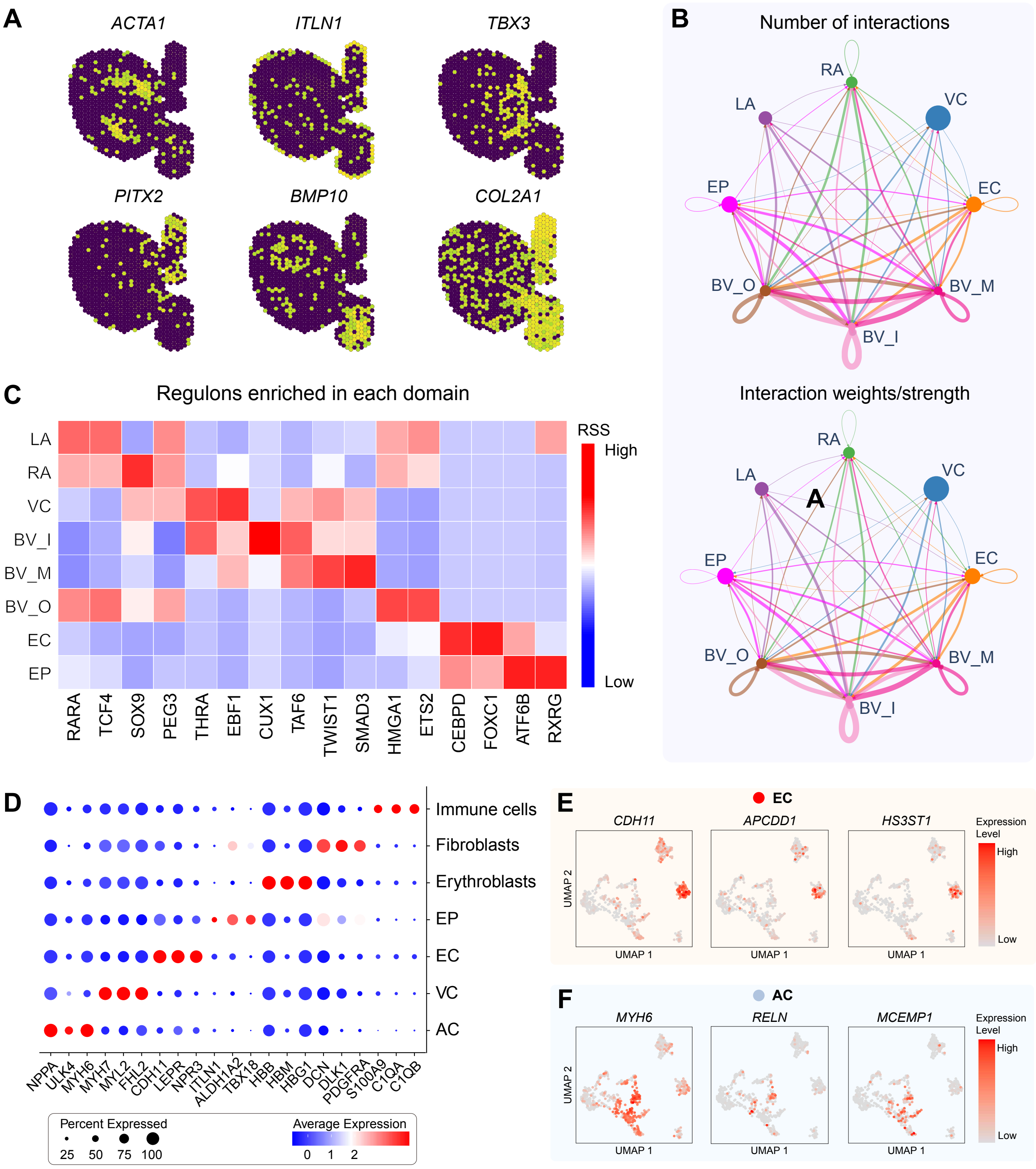

A
ACTA1
ITLN1
TBX3
PITX2
BMP10
COL2A1
B
Number of interactions
RA
LA
VC
EP
EC
BV_O
BV_M
BV_I
Interaction weights/strength
C
Regulons enriched in each domain
LA
RA
VC
BV_I
BV_M
BV_O
EC
EP
RSS
High
Low
RARA
TCF4
SOX9
PEG3
THRA
EBF1
CUX1
TAF6
TWIST1
SMAD3
HMGA1
ETS2
CEBPD
FOXC1
ATF6B
RXRG
D
Immune cells
Fibroblasts
Erythroblasts
EP
EC
VC
AC
NPPA
ULK4
MYH6
MYH7
MYL2
FHL2
CDH11
LEPR
NPR3
ITLN1
ALDH1A2
TBX18
HBB
HBM
HBG1
DCN
DLK1
PDGFRA
S100A9
C1QA
C1QB
Percent Expressed
25 50 75 100
Average Expression
0 1 2
E
EC
CDH11
APCDD1
HS3ST1
Expression Level
High
Low
UMAP 2
UMAP 1
F
AC
MYH6
RELN
MCEMP1

Supplementary Figure 17. GSG identifies spatial domains in human fetal heart ST data. A. Spatial expression of six marker genes of different domains. **B.** Spatial domain communication network analysis shows that the blood vessel and epicardium domains are the main sources of signaling. The edge colors correspond to the signaling source, and the circle sizes in upper and bottom parts of the plot indicate the interaction number and strength, respectively. **C.** Regulon analysis by SCENIC indicates regulons enriched in each spatial domain. **D.** The dot plot displays the marker gene expression profiles of the seven cell types in 9-13 weeks of fetal human hearts. **E.** Marker gene expression level of EC cells in UMAP embeddings. **F.** Marker gene expression level of AC cells in UMAP embeddings.

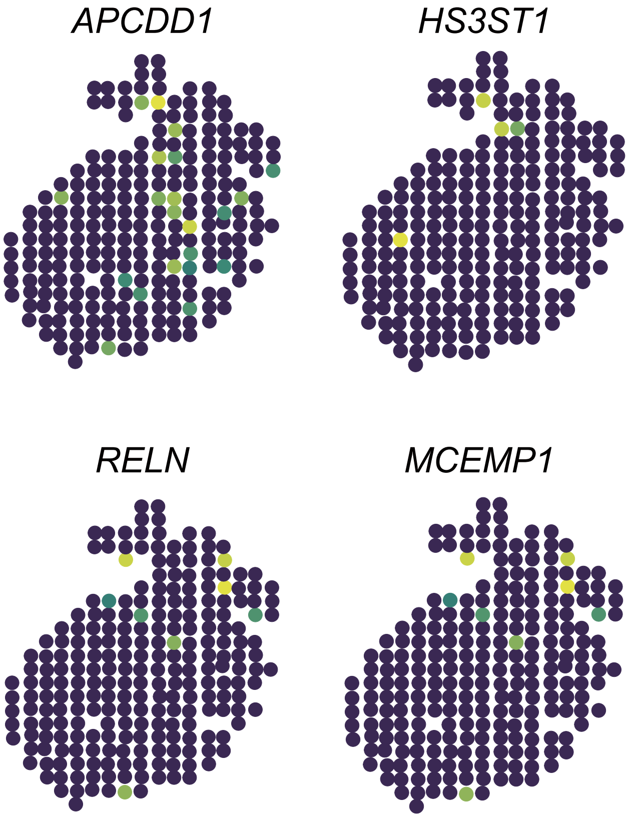

Supplementary Figure 18. Spatial expression of four genes expressed in the fetal human heart (9 weeks of gestation) ST published before that exhibit low quality compared to Fig. 4D.

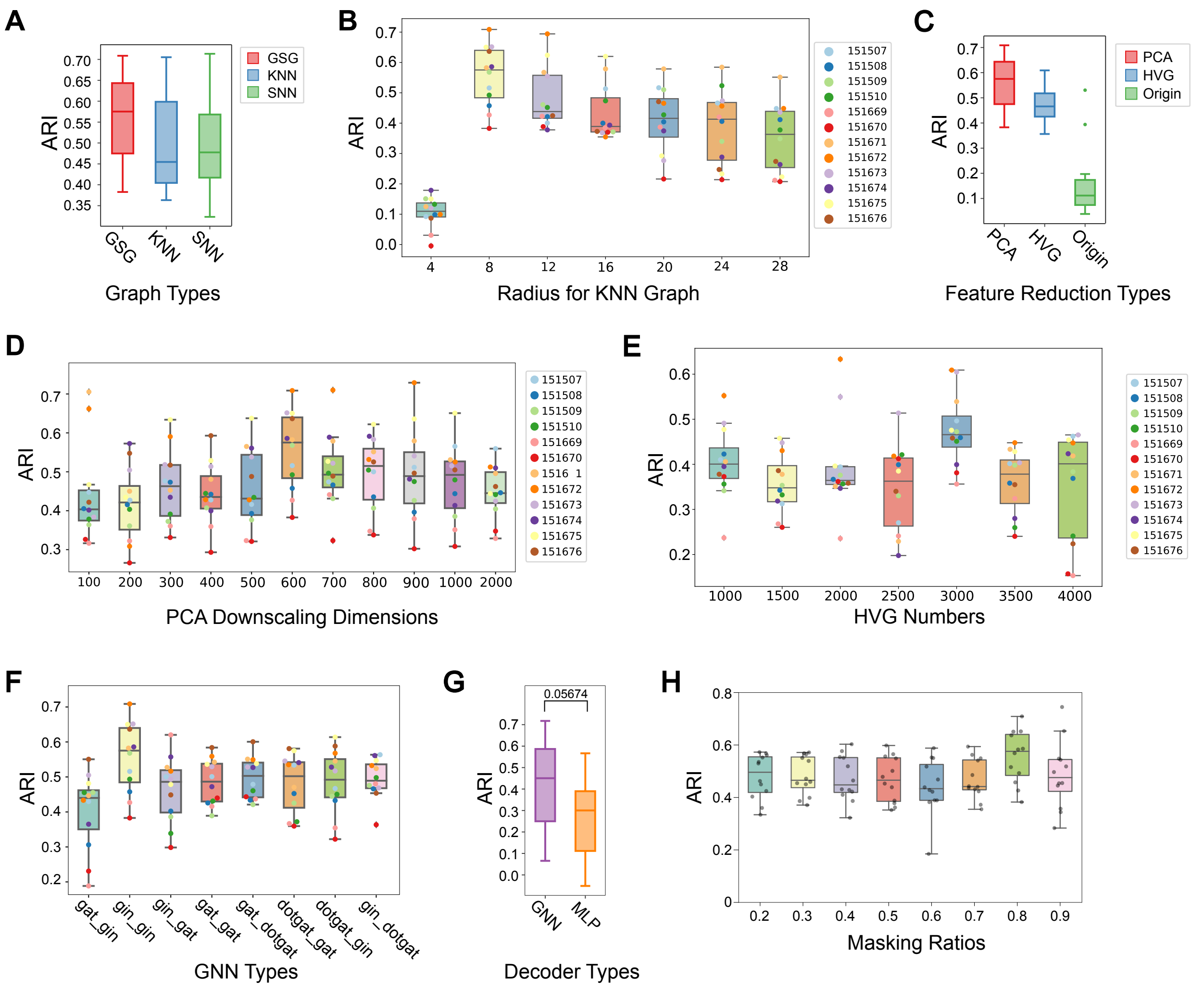

Supplementary Figure 19. Sensitivity analysis of GSG on DLPFC datasets. A. Boxplots showing the ARI scores of three methods for constructing adjacency matrices in GSG. **B.** ARI boxplots comparing different KNN radius for constructing adjacency matrix in GSG. **C.** Boxplots showing the ARI scores with or without feature selection using two different methods, PCA and HVG. **D.** The ARI pirate graph of PCA reduced dimensions of ST data in GSG. **E.** The ARI boxplots of numbers of HVG extracted genes of ST data in GSG. **F.** The ARI boxplots of nine different GNN types, each evaluated on 12 DLPFC slides. **G.** Boxplots comparing decoder types in GSG based on ARI scores. **H.** Boxplots illustrating the ARI scores of GSG under varying masking ratios. In each boxplot, the center line, box limits, and whiskers represent the median, upper/lower quartiles, and 1.5× the interquartile range, respectively.

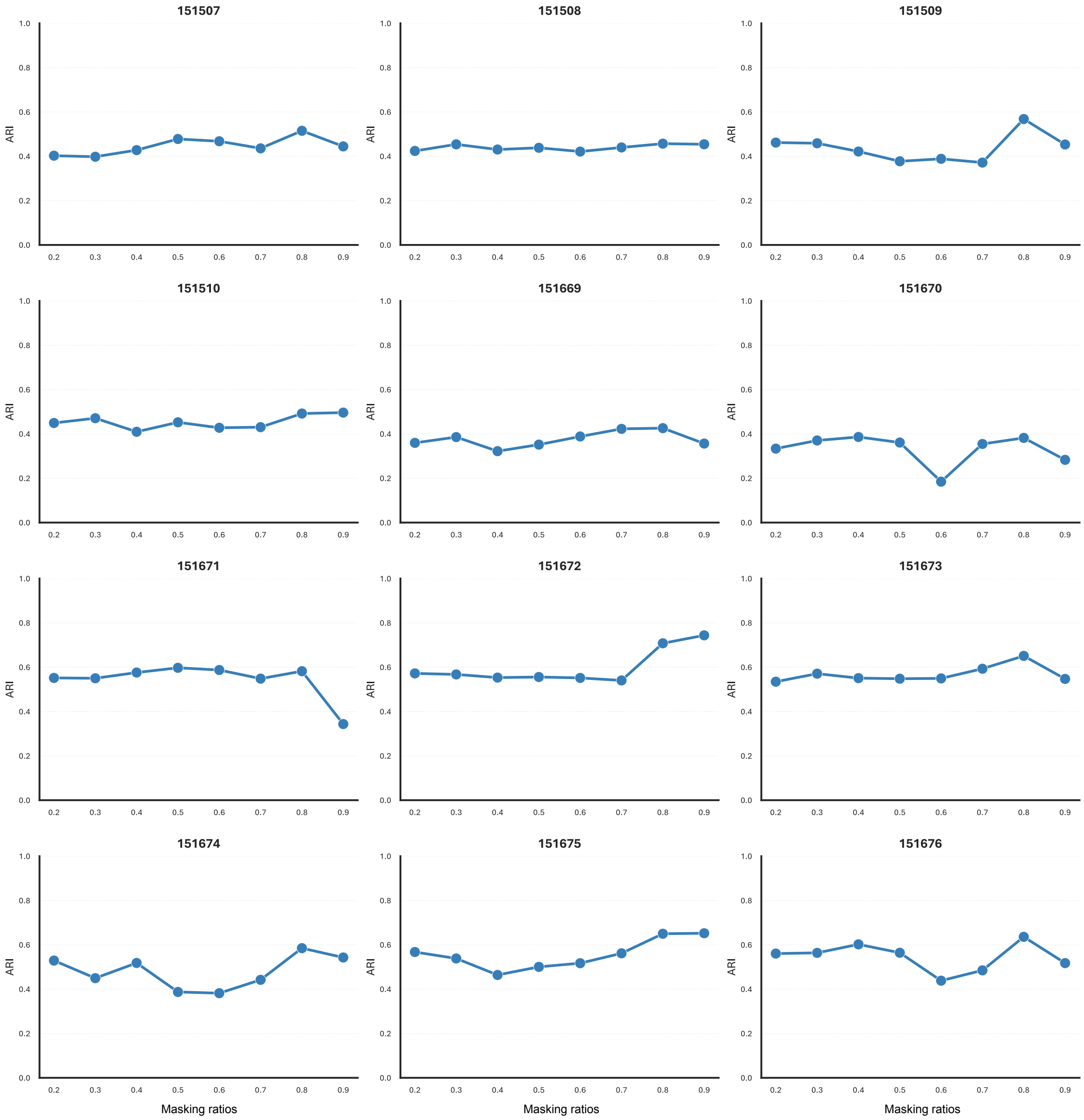

Supplementary Figure 20. Sensitivity analysis of the masking ratio across 12 DLPFC slices. Each sub-panel displays the ARI scores achieved on an individual slice as the masking ratio varies from 0.2 to 0.9.

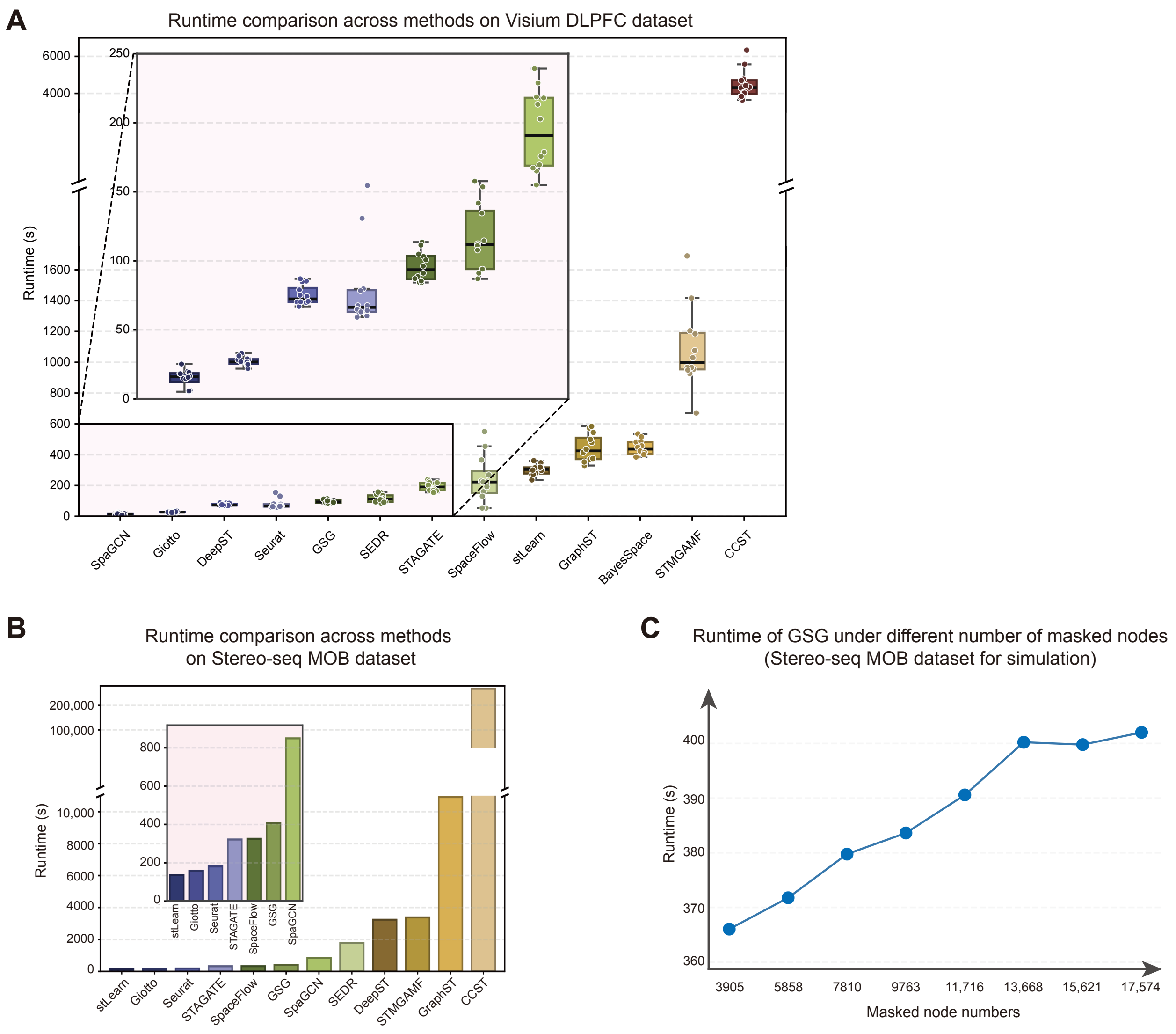

Supplementary Figure 21. Computational efficiency of GSG. A. Boxplot of runtime of GSG and baseline models on 12 slices in DLPFC dataset. Each point represents a slice. In each boxplot, the center line, box limits, and whiskers represent the median, upper, and lower quartiles, and 1.5× inter-quartile range, respectively. **B.** Bar plot of runtime of GSG and baseline models on Stereo-seq mouse olfactory bulb (MOB) dataset. **C.** Line plot of the runtime of GSG under different number of masked nodes, simulated on Stereo-seq mouse olfactory bulb dataset.

References

S. Aibar, C. B. González-Blas, T. Moerman, et al. Scenic: single-cell regulatory network inference and clustering. *Nat. Methods*, 14(11):1083–1086, 2017.

K. Dong and S. Zhang. Deciphering spatial domains from spatially resolved transcriptomics with an adaptive graph attention auto-encoder. *Nat. Commun.*, 13(1):1739, 2022.

R. Dries, Q. Zhu, R. Dong, et al. Giotto: a toolbox for integrative analysis and visualization of spatial expression data. *Genome Biol.*, 22:1–31, 2021.

Y. Fu, M. Nan, Q. Ren, et al. A multi-view graph convolutional network framework based on adaptive adjacency matrix and multi-strategy fusion mechanism for identifying spatial domains. *Bioinformatics*, 41(4):btaf172, 04 2025.

Y. Hao, S. Hao, E. Andersen-Nissen, et al. Integrated analysis of multimodal single-cell data. *Cell*, 184(13): 3573–3587, 2021.

J. Hu, X. Li, K. Coleman, et al. Spagcn: Integrating gene expression, spatial location and histology to identify spatial domains and spatially variable genes by graph convolutional network. *Nat. Methods*, 18 (11):1342–1351, 2021.

S. Jin, C. F. Guerrero-Juarez, L. Zhang, et al. Inference and analysis of cell-cell communication using cellchat. *Nat. Commun.*, 12(1):1088, 2021.

R. S. King and P. A. Newmark. In situ hybridization protocol for enhanced detection of gene expression in the planarian schmidtea mediterranea. *BMC Dev. Biol.*, 13:1–16, 2013.

J. Li, S. Chen, X. Pan, et al. Cell clustering for spatial transcriptomics data with graph neural networks. *Nat. Comput. Sci.*, 2(6):399–408, 2022.

Y. Long, K. S. Ang, M. Li, et al. Spatially informed clustering, integration, and deconvolution of spatial transcriptomics with graphst. *Nat. Commun.*, 14(1):1155, Mar 2023.

K. R. Maynard, L. Collado-Torres, L. M. Weber, et al. Transcriptome-scale spatial gene expression in the human dorsolateral prefrontal cortex. *Nat. Neurosci.*, 24(3):425–436, 2021.

L. McInnes, J. Healy, and J. Melville. Umap: Uniform manifold approximation and projection for dimension reduction. *arXiv*, 2018. https://doi.org/10.48550/arXiv.1802.03426.

B. J. Pearson, G. T. Eisenhoffer, K. A. Gurley, et al. Formaldehyde-based whole-mount in situ hybridization method for planarians. *Dev. Dyn.*, 238(2):443–450, 2009.

F. Pedregosa, G. Varoquaux, A. Gramfort, V. Michel, et al. Scikit-learn: Machine learning in python. *J. Mach. Learn. Res.*, 12:2825–2830, 2011.

D. Pham, X. Tan, B. Balderson, et al. Robust mapping of spatiotemporal trajectories and cell–cell interactions in healthy and diseased tissues. *Nat. Commun.*, 14:7739, 2023.

H. Ren, B. L. Walker, Z. Cang, et al. Identifying multicellular spatiotemporal organization of cells with spaceflow. *Nat. Commun.*, 13(1):4076, 2022.

F. A. Wolf, P. Angerer, and F. J. Theis. Scanpy: large-scale single-cell gene expression data analysis. *Genome Biol.*, 19:1–5, 2018.

F. A. Wolf, F. K. Hamey, M. Plass, et al. Paga: graph abstraction reconciles clustering with trajectory inference through a topology preserving map of single cells. *Genome Biol.*, 20:1–9, 2019.

C. Xu, X. Jin, S. Wei, et al. Deepst: identifying spatial domains in spatial transcriptomics by deep learning. *Nucleic Acids Res.*, 50(22):e131–e131., 2022.

H. Xu, H. Fu, Y. Long, et al. Unsupervised spatially embedded deep representation of spatial transcriptomics. *Genome Med.*, 16:12., 2024.

E. Zhao, M. R. Stone, X. Ren, et al. Spatial transcriptomics at subspot resolution with bayesspace. *Nat. Biotechnol.*, 39(11):1375–1384, 2021.
